# A dual-caged resorufin probe for rapid screening of infections resistant to lactam antibiotics†

**DOI:** 10.1039/d1sc01471d

**Published:** 2021-05-28

**Authors:** Jinghang Xie, Ran Mu, Mingxi Fang, Yunfeng Cheng, Fiona Senchyna, Angel Moreno, Niaz Banaei, Jianghong Rao

**Affiliations:** Departments of Radiology and Chemistry, Molecular Imaging Program at Stanford, Stanford University School of Medicine Stanford CA 94305 USA jrao@stanford.edu; Department of Pathology, Stanford University School of Medicine Stanford CA 94305 USA; Clinical Microbiology Laboratory, Stanford University Medical Center Palo Alto CA 94304 USA; Division of Infectious Diseases and Geographic Medicine, Department of Medicine, Stanford University School of Medicine Stanford CA 94305 USA

## Abstract

The alarming increase of antimicrobial resistance urges rapid diagnosis and pathogen specific infection management. This work reports a rapid screening assay for pathogenic bacteria resistant to lactam antibiotics. We designed a fluorogenic N-cephalosporin caged 3,7-diesterphenoxazine probe CDA that requires sequential activations to become fluorescent resorufin. A series of studies with recombinant β-lactamases and clinically prevalent pathogens including *Escherichia coli*, *Klebsiella pneumoniae*, *Enterobacter cloacae* and *Serratia marcescens* demonstrated that CDA possessed superior sensitivity in reporting the activity of β-lactamases including cephalosporinases and carbapenemases. After a simple filtration, lactam-resistant bacteria in urine samples could be detected at 10^3^ colony-forming units per milliliter within 2 hours.

## Abstract 2

This work reports a dual-caged fluorogenic probe and its demonstration for rapid and sensitive screening of as low as 1000 clinically isolated β-lactamase-expressing bacteria per milliliter urine samples within 2 hours.

## Graphical abstract

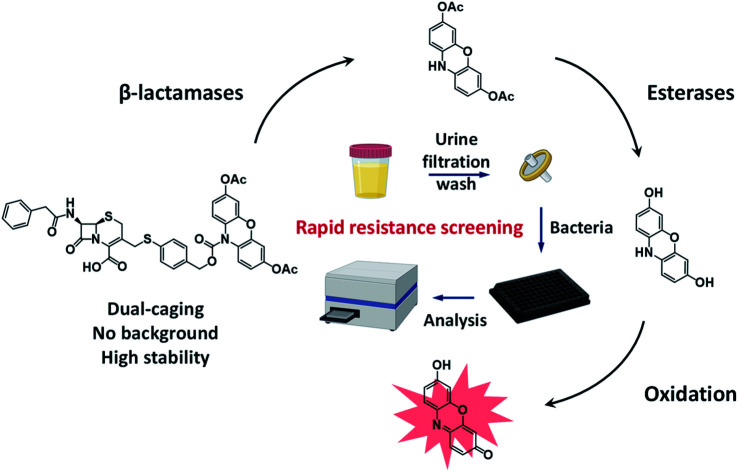

Superbugs could kill as many as 10 million people each year globally by 2050, nearly 2 million more than cancer.^1^ The lack of pre-prescription antimicrobial susceptibility testing (AST) that can inform the burden of drug resistant pathogenic bacteria, such as rapid and accurate diagnosis to identify common resistance mechanisms, has increased unnecessary antimicrobial usage, delayed pathogen-specific infection management, and allowed continued spreading of drug resistance.

Discovered nearly a century ago, β-lactam antibiotics (*e.g.*, penicillins, cephalosporins and carbapenems) remain the most successful class of antimicrobials, constituting 60% of worldwide antibiotic usage, and are among the most effective agents for treatment of infections.^2^ One major acquirable mechanism for β-lactam resistance is the production of β-lactamases which break down β-lactams to metabolites incapable of binding to PBPs (penicillin-binding proteins). From 2010 to 2014, the rates of extended-spectrum β-lactamase (ESBL)-positive *Escherichia coli* (*E. coli*) isolated in urinary specimens from across the United States increased from 7.9 to 18.3%; the activity of all tested cephalosporin antibiotics decreased significantly by about 9%.^3^ More recent studies revealed a surging rate of ESBL-producing Enterobacterales in the US, Europe, and China from 30% to 49.4%.^4–7^ Isolates carrying carbapenemases, the enzymes that could hydrolyze nearly all β-lactam antibiotics including broad-spectrum carbapenem antibiotics, are also on a rising trend.^3^ Development of a rapid screening assay for β-lactamase activity is critical for combating resistance to lactam antibiotics and enhancing antimicrobial stewardship.

Genotypic nucleic acid testing (NAT) like FISH (fluorescence *in situ* hybridization) and PCR have been used for expeditious detection of β-lactamase genes. But NAT has some major limitations. First, it is nearly impossible to develop a NAT that can cover all β-lactamase genes and mutations, considering that more than one thousand distinct β-lactamases have been identified in natural clinical isolates.^8^ Second, the detection of nucleic acid does not predict the persistence of viable pathogens, as the inactivation of pathogens by antibiotics often triggers a slow decay of cellular components including cytoplasmic membranes.^9,10^ Third, the mere presence of genetic materials does not necessarily imply their expression or function and poses significant challenges in typing strains or isolates with real antimicrobial resistance and toxin production.^10,11^ Emerging diagnostic platforms are employing microfluidics, biosensors, and lab-on-a-chip technology for genotypic and phenotypic detection of β-lactamases.^12–14^ Rapid phenotypic ASTs hybridized with microfluidic chips and biosensors measuring optical,^15^ chemical,^16–18^ electrical,^19–22^ mechanical,^23,24^ or other^25,26^ signals during bacterial growth in the presence of β-lactam antibiotics have been demonstrated even with very low numbers of bacteria and sample volumes and in parallel testing of different pathogens with multiple antibiotics. While holding great potential, their robustness, cost-effectiveness, and adaptability to large scale clinical settings remain to be further illustrated.

Conventional phenotypic AST remains the diagnostic standard for reporting bacterial infections resistant to β-lactam antibiotics, although it requires considerable effort in designated laboratories and takes at least 1–2 days to finish.^27^ Chromogenic assays such as the Cefinase test and β-Lacta could be performed before AST to report the activity of broad spectrum β-lactamases, but initial cultural enrichment is indispensable.^28,29^ Recent development of a papain-mediated amplification strategy allowed detection of β-lactamase activity in raw patient specimens like urine, yet gave a limit of detection at around 10^5^ to 10^6^ colony-forming units (cfu) in a 96 well plate.^30^ Fluorescence-based bioanalytical assays offer advantages such as high sensitivity, ease of use, and low cost. Some fluorogenic probes for the detection of β-lactamase activity in bacteria have been described with mainly coumarin and fluorescein analogues as reporters, including those synthesized by us such as CDC, CDG, and (S)-CC series of compounds.^31–37^ Nevertheless, the autofluorescence in patient specimens ranging from violet to green and yellow remains a major roadblock for direct sample analysis with these probes.^38–40^ Attempts have been made to circumvent the issue by using redshift fluorophores. The β-LEAF assay, for instance, employed a unique quenching–dequenching mechanism with benzo[*a*]phenothiazines and could generate red fluorescence (excitation, 640 nm/emission, 700 nm) upon β-lactamase activation. However, the assay still needs cultured bacteria, probably due to a moderate sensitivity.^41^ Other near-infrared probes were mainly designed for imaging β-lactamases as a reporter in mammalian cells with a fluorescence microscope or tracking infections like tuberculosis *in vivo*.^42–45^ The overall sensitivity of existing probes still needs to be improved for direct analysis of patient samples without culture. Therefore, development of new caging strategies with red fluorophores may address these shortcomings and fulfill the need for a rapid and sensitive screening assay of β-lactamase activity within a few hours of sample collection, especially at acute care clinics.

Here, we report a dual-caging design of a novel N-cephalosporin caged 3,7-diesterphenoxazine probe named CDA (Cephalosporin caged Diester Amplex red analogue) (Fig. 1a) and the development of a fluorogenic assay for detecting β-lactamase expressing bacteria within hours of sample collection. As a highly fluorescent reporter (*Φ* = 0.75, pH = 8), resorufin shows excitation/emission at ∼570/585 nm, and could be caged for sensing enzyme activity,^46^ such as the N-protected 3,7-dihydroxyphenoxazine^47–50^ and *O*-alkyl resorufin derivatives.^31,51,52^ As a precursor of a fluorescent probe, the amine protected phenoxazine has overall low fluorescence background, thus affording a high turn-on ratio.^47^ 10-Acetyl-3,7-dihydroxyphenoxazine (Amplex Red), for example, takes advantage of naturally low autofluorescence in biological samples and could determine hydrogen peroxide (H_2_O_2_) at nano- to picomolar levels. In this study, we caged the amine with an analogue of cefazolin, the first generation cephalosporin antibiotic, and the substrate of a broad spectrum of β-lactamases from cephalosporinases to carbapenemases. Esterification of the 3,7-hydroxy groups allowed better uptake by Gram-negative bacteria and minimized its background fluorescence.^53^ CDA could traverse the capsule either through passive diffusion, or through active porin uptake,^54^ and then encounters β-lactamases within the periplasm of resistant bacteria where the cephalosporin moiety would be hydrolyzed to generate reduced diester resorufin (DARR). Membrane permeable DARR would be further hydrolyzed by universally present esterases in viable bacteria followed by oxidation to produce highly fluorescent resorufin (Fig. 1a). Without β-lactamases, CDA cannot be converted to a fluorescent product by esterases alone (Fig. 1b). Compared to singly caged fluorogenic probes, the cascade activation mechanism of CDA should allow lower background and promise better detection sensitivity.

**Fig. 1:**
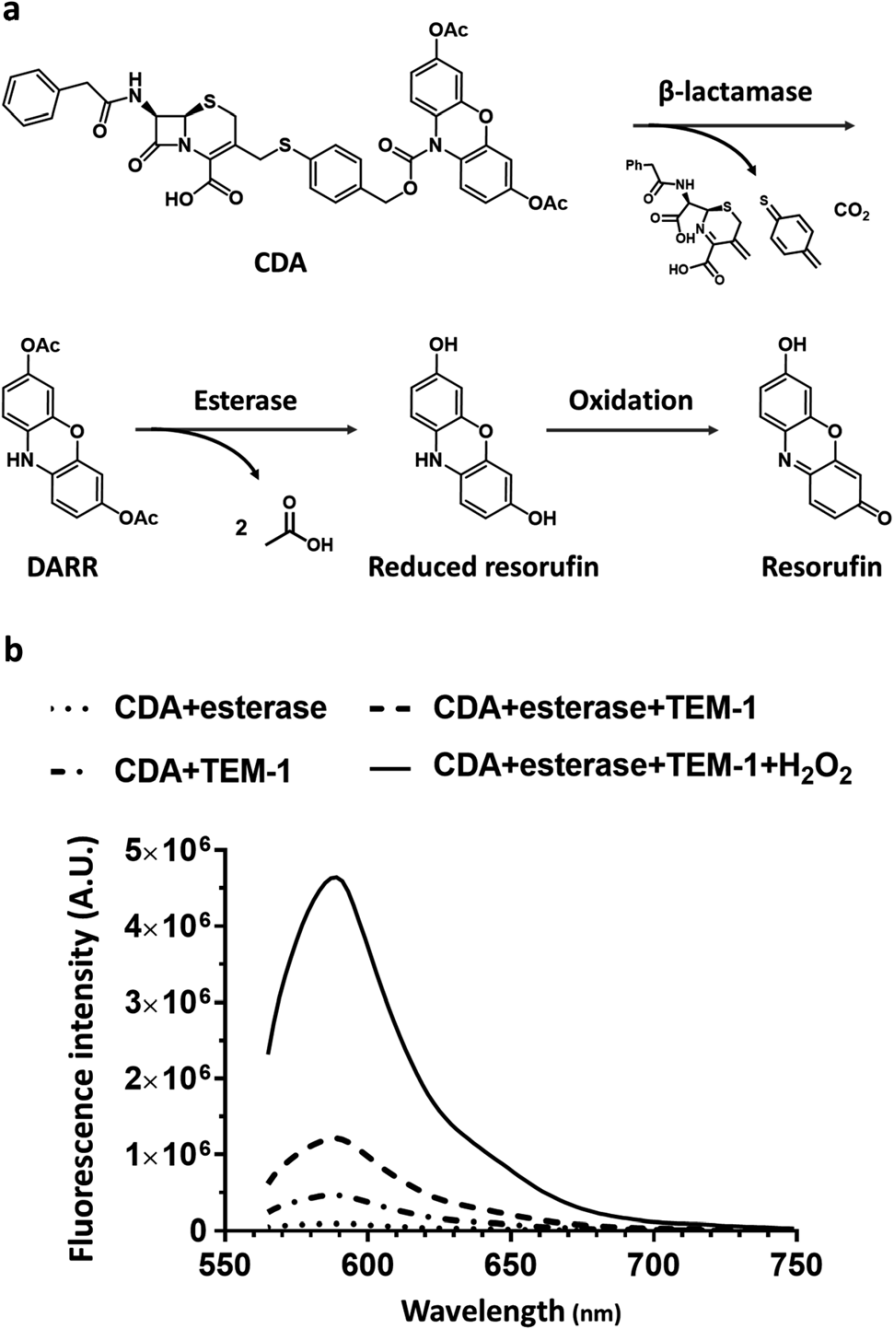
The design of CDA for targeting cephalosporinases and carbapenemases. (a) Structure of CDA and its hydrolysis by β-lactamase (Bla) and esterase, followed by oxidation to yield fluorescent resorufin. (b) Fluorescence emission spectra (565–750 nm) of CDA, and CDA treated with esterase (1 μg mL^−1^), TEM-1 Bla (100 nM), and both enzymes with and without 1 mM H_2_O_2_ at room temperature for 8 hours (excitation, 525 nm). The activation of CDA results in a 1200-fold increase for emission at 585 nm over CDA in PBS. Spectra were collected on a SpectraMax iD3 multimode microplate reader. A.U. indicates arbitrary units.

The synthesis of CDA is outlined in Scheme 1. DARR was prepared from resazurin sodium salt through reduction and acetylation^47^ and conjugated to the oxidized cephalosporin chloride with a self-immolative linker 4-mercaptobenzyl alcohol to afford CDA after reduction and deprotection. When incubated with a recombinant class A cephalosporin-hydrolyzing β-lactamase TEM-1 (TEM-1 Bla, Fig. S1 and S2†),^33,55^ esterase, and hydrogen peroxide (H_2_O_2_), CDA generated ∼1200-fold fluorescence turn-on (Fig. 1b and S3†). In comparison, Amplex Red with 3,7-hydroxyls showed initially higher fluorescence and 413-fold turn-on in the presence of horseradish peroxidase (HRP) and H_2_O_2_ (Fig. S4†).

**Scheme 1:**
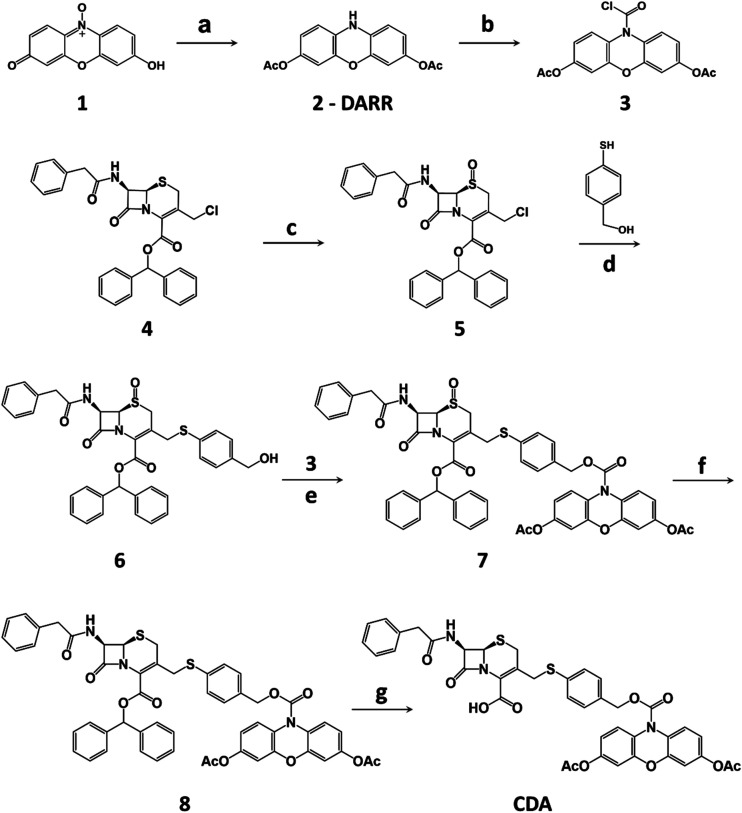
Synthesis of CDA. (a) (i) Zn/AcOH, RT; (ii) acetone, DMAP, Ac_2_O, RT. (b) (i) Triphosgene/TEA; (ii) DCE. (c) *m*CPBA, DCM, 0 °C to RT. (d) K_2_CO_3_, CH_3_CN. (e) K_2_CO_3_/DMAP, DCE, Ar, RT. (f) NaI/TFAA, acetone, 0 °C, Ar. (g) TFA/TIPS, DCM, RT. RT = room temperature, DMAP = 4-dimethylaminopyridine, TEA = triethanolamine, *m*CPBA = *meta*-chloroperoxybenzoic acid, TFAA = trifluoroacetic anhydride, DCE = 1,2-dichloroethane, TFA = trifluoroacetic acid, DCM = dichloromethane, TIPS = triisopropylsilane.

As oxidation is an essential step in converting β-lactamase/esterase processed CDA to resorufin, we evaluated the stability of CDA in different concentrations of H_2_O_2_ (Fig. 2a). Nearly no fluorescence turn-on was observed in the presence of up to 1 mM H_2_O_2_ in PBS within 2 hours of incubation at room temperature. The sulfur center of CDA was also stable in H_2_O_2_ (1 mM) (Fig. S5†). In contrast, DARR was less stable and presented concentration-dependent turn-on in H_2_O_2_ and fast response to esterase (Fig. S6 and S7†). CDA treated with TEM-1 Bla and esterase showed fluorescence turn-on without H_2_O_2_ (Fig. 2b), because the dissolved oxygen in solution efficiently oxidized the TEM-1 Bla/esterase processed CDA into resorufin (Fig. 2c). Either H_2_O_2_ alone at a concentration above 1 mM or H_2_O_2_ (∼1 μM) with HRP (0.1–1 unit per mL) could significantly enhance the fluorescence turn-on (Fig. 2d and S8†). Under our current conditions, 100 nM CDA at the minimum is required for reporting β-lactamase/esterase activity and 10 μM CDA generated ideal signal strength (Fig. 2e). Esterases alone barely changed the response of CDA to H_2_O_2_ (Fig. S9†). To demonstrate the ability of CDA in detecting a broad range of β-lactamases, we expressed and purified several clinically prevalent β-lactamases in addition to TEM-1 Bla over all four Ambler classes: extended spectrum AmpC (class C), *Mycobacterium tuberculosis* specific BlaC (class A), and carbapenemases IMP-1 (class B), OXA-48 (class D) and KPC-3 (class A) (Fig. S10–S15†). CDA could detect all β-lactamases as low as 1 femtomole in 2 hours at room temperature, a sensitivity unattainable with previously developed β-lactamase fluorogenic probes (Fig. 2f).^33^ Here, the limit of detection was determined by the fluorescent signal equal to three times the standard deviation of the negative controls: CDA in 1 mM H_2_O_2_/PBS with esterase at room temperature for 2 hours. Together, these data suggest a good sensitivity and selectivity of CDA towards β-lactamases.

**Fig. 2:**
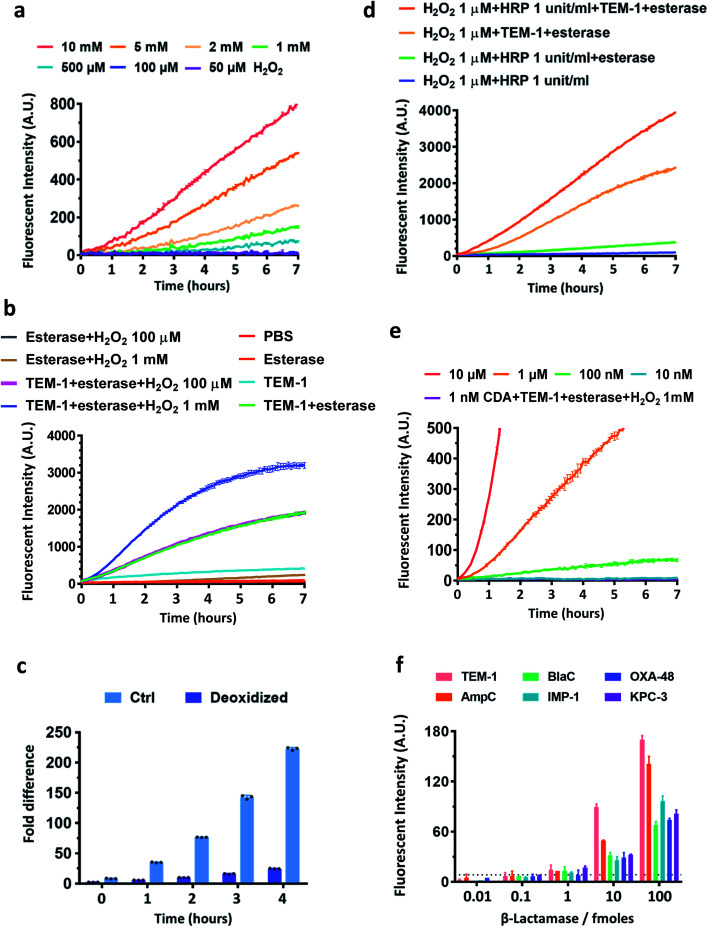
Characterization of CDA with recombinant β-lactamases, esterase, HRP, and H_2_O_2_. (a) Stability test of CDA in different concentrations of H_2_O_2_ in PBS. The signal of PBS was subtracted and the absolute values were plotted. (b) Fluorescence enhancement of CDA treated with esterase (1 μg mL^−1^), TEM-1 Bla (100 nM) and H_2_O_2_ (100 μM or 1 mM). (c) Activation of CDA by esterase (1 μg mL^−1^) and TEM-1 Bla (100 nM) with or without oxygen in the reaction. Glass flasks with working solution were continuously vacuumed to maintain a deoxidized environment. Samples were transferred and scanned immediately at each time point in a black 96 well plate. Error bars represent ± SD, *n* = 3. (d) Fluorescence enhancement of CDA treated with esterase (1 μg mL^−1^), TEM-1 Bla (100 nM), HRP (1 unit per mL), and H_2_O_2_ (1 μM). (e) Fluorescence enhancement of different concentrations of CDA treated with esterase (1 μg mL^−1^), TEM-1 Bla (100 nM) and H_2_O_2_ (1 mM). (f) Fluorescence enhancement of CDA treated with indicated amounts of β-lactamases, esterase (1 μg mL^−1^) and H_2_O_2_ (1 mM) after incubation at room temperature for 2 h. The dashed line represents 3 times the standard deviation of the mean value of the negative control (PBS). All the studies were duplicated at room temperature in PBS (pH = 7.05) as the buffer unless otherwise indicated. The working concentration of CDA was 10 μM except (e). Data were collected with excitation at 570 nm and emission at 600 nm. A.U. indicates arbitrary units.

Next, we studied if CDA could report β-lactamase activity in live *E. coli* and *Klebsiella pneumoniae* (*K. pneumoniae*) which are commonly found in many infections.^3,56^ Three transformed *E. coli* strains expressing the most clinically prevalent β-lactamases including TEM-1 Bla (*E. coli*/TEM-1) and two carbapenemases were evaluated: a metallo-β-lactamase IMP-1 (*E. coli*/IMP-1) and a unique class A β-lactamase KPC-3 (*E. coli*/KPC-3) that hydrolyze carbapenem antibiotics.^33^ The number of bacteria in the assay was verified by calibrating the optical density measurement at 600 nm (OD_600_) and bacterial colony-forming units (Fig. S16†). The viability and integrity of freshly cultured bacteria were confirmed with fluorescein diacetate and propidium iodide staining with heat-killed bacteria as a control (Fig. S17†). Because CDA could be activated by TEM-1 Bla and esterase in PBS without extra oxidative reagents, we first explored whether CDA alone could detect *E. coli*/TEM-1. As shown in Fig. 3a, to a limited extent, only a large number of TEM-1 Bla producing bacteria was detected (10^7^ cfu mL^−1^) (Fig. S18a†), suggesting that the level of H_2_O_2_ or oxygen within bacteria may not be sufficient to oxidize the hydrolyzed product to resorufin.^57,58^ The addition of HRP and H_2_O_2_ (1 μM) contributed little to the fluorescence increase unless bacteria were lysed (Fig. 3b). As HRP has no means to penetrate cell membranes, these results indicated that the reduced resorufin was mostly retained intracellularly and 1 μM H_2_O_2_ was not efficient in oxidizing it to resorufin. Many studies have reported the high permeability of *E. coli* membranes to environmental H_2_O_2_.^59,60^ Would a higher concentration of H_2_O_2_ equilibrate across membranes of viable bacteria and accelerate the oxidation of processed CDA? To address this question, a serial dilution of H_2_O_2_ (100 μM, 500 μM, 1 mM, 2 mM) was incubated with CDA and different concentrations of *E. coli* and *E. coli*/TEM-1 for a longitudinal reading (Fig. 3c, S18b and S19†). With 1 mM H_2_O_2_/CDA, 10^5^ cfu mL^−1^ of *E. coli*/TEM-1 were detected within 2–3 hours and 10^4^ cfu mL^−1^ after 4 hours of incubation at room temperature. A longer preincubation (∼20 hours) with CDA before H_2_O_2_ addition could detect as low as 10^3^ cfu mL^−1^ (Fig. 3d). Factoring the 100 μL assay volume, approximately 100 bacteria in one 96 well were detectable with this assay. To evaluate the role of the 3,7-diacetate of CDA, we preincubated CDA with esterase to fully hydrolyze both esters before incubation with *E. coli*/TEM-1 and H_2_O_2_. A much higher initial fluorescence intensity and a smaller fold increase were observed which confirmed the critical role of the diester in lowering background and enhancing sensitivity (Fig. S20†).

**Fig. 3:**
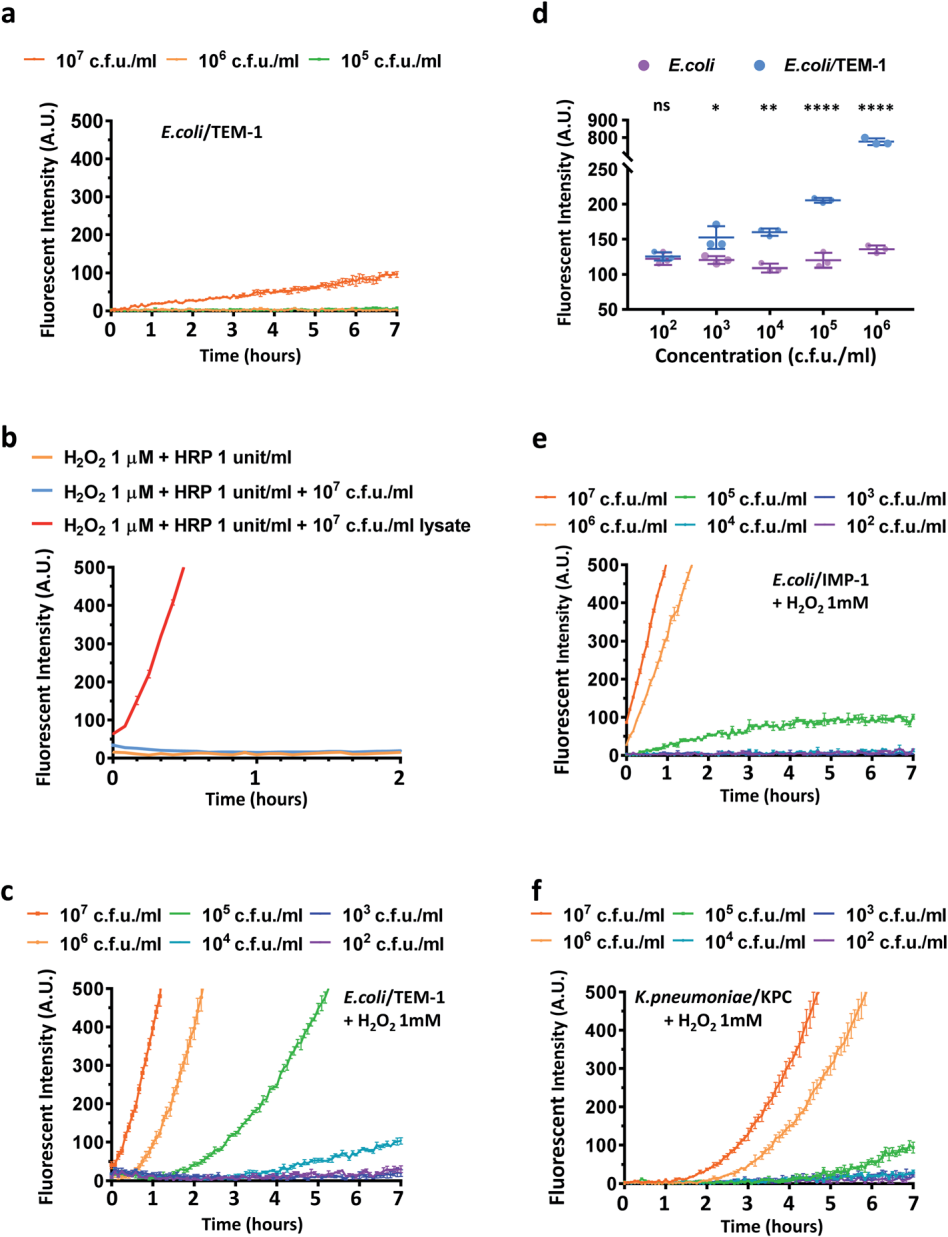
Characterization of CDA with HRP, H_2_O_2_, *E. coli* expressing TEM-1 Bla (*E. coli*/TEM-1) and IMP-1 carbapenemase (*E. coli*/IMP-1), and *K. pneumoniae* expressing KPC-type carbapenemase (*K. pneumoniae*/KPC). (a) Fluorescence enhancement of CDA at different concentrations of *E. coli* expressing TEM-1 Bla. (b) Fluorescence enhancement of CDA; HRP (1 unit per mL), H_2_O_2_ (1 μM), and either viable *E. coli* expressing TEM-1 Bla or bacterial lysate extracted from equal cfu per milliliter. (c) Fluorescence enhancement of CDA and different concentrations of *E. coli* expressing TEM-1 Bla (H_2_O_2_, 1 mM). (d) Fluorescence intensity of *E. coli* and *E. coli*/TEM-1 preincubated with CDA for 20 hours upon addition of H_2_O_2_ (500 μM) for 5 min. Error bars represent ± SD, *n* = 3. *p < 0.0332; **p < 0.0021, ****p < 0.0001, ns: not significant. (e) Fluorescence enhancement of CDA at different concentrations of *E. coli* expressing IMP-1 carbapenemase in the presence of H_2_O_2_ (1 mM). (f) Fluorescence enhancement of CDA at different concentrations of *K. pneumoniae* expressing KPC-type carbapenemase (H_2_O_2_, 1 mM). All the studies were duplicated at room temperature with PBS (pH = 7.05) as the buffer unless otherwise indicated. The working concentration of CDA was 10 μM. The signal of CDA in PBS (a), H_2_O_2_, 1 μM (b), or H_2_O_2_, 1 mM (c, e, f) was subtracted and the absolute values were plotted. Data were collected on a Tecan Safire microplate reader with excitation at 570 nm and emission at 600 nm. A.U. indicates arbitrary units.

Next, the assay was used to detect *E. coli*/IMP-1 and *E. coli*/KPC-3 (Fig. 3e and S21†). *E. coli*/IMP-1 was detected at 10^5^ cfu mL^−1^ within 2 hours, and *E. coli*/KPC-3 could be reliably detected at around 10^5^ to 10^6^ cfu mL^−1^. As KPC-3 carbapenemase has shown comparable activity to TEM-1 Bla in hydrolyzing cephalosporin substrates,^33^ we investigated whether the expression level of β-lactamases contributed to the variation in detection sensitivity. When TEM-1 Bla transformed *E. coli* were not induced (Fig. S22†), the fluorescence turn-on was compromised due to low expression of TEM-1 Bla, but 10^6^ to 10^7^ cfu mL^−1^ of bacteria were still detectable within 2–3 hours.

We further applied CDA for detecting *K. pneumoniae*. Two clinical isolates either imipenem susceptible or resistant by expressing a KPC-type carbapenemase (*K. pneumoniae*/KPC) confirmed in a previous clinical study were incubated with CDA and H_2_O_2_ (Fig. 3f and S23†).^61^ The number of *K. pneumoniae* in the assay was verified and OD_600_ of 1.0 gave roughly 1 × 10^9^ cfu mL^−1^ of bacteria (Fig. S24†). Similar to *E. coli*/KPC-3, 10^6^ cfu mL^−1^ of KPC-positive *K. pneumoniae* could be detected within 2–3 hours. Collectively, these data suggested that the CDA/H_2_O_2_ assay may be useful for detecting cephalosporinase- and carbapenemase-expressing bacteria in clinics.

To develop an easy-to-use, rapid assay for screening cephalosporinase and carbapenemase activity, we employed a simple two-step filtration method with common laboratory supplies (Fig. S25†) to prepare and concentrate pathogenic bacteria. A similar procedure has been applied for improving culture-based AST previously.^62^ As shown in Fig. 4a, either processed or unprocessed patient samples will be filtered sequentially through 5 micron and 0.22 micron syringe filters. The 5 micron filter would trap particles including cells and large debris, and bacteria would be collected by the 0.22 micron filter and washed with PBS. A reverse elution with PBS containing 1 mM H_2_O_2_ allowed immediate high-throughput analysis by a plate reader in a 96-well plate precoated with CDA. Synthetic urine samples were used as a model to test the workflow: 50 mL of synthetic urine spiked with bacteria could be concentrated to an ∼200 μL ready-to-use suspension within a few minutes; polypropylene filters recover bacteria more efficiently (>90%) than commonly used nylon filters (Fig. S26†).

**Fig. 4:**
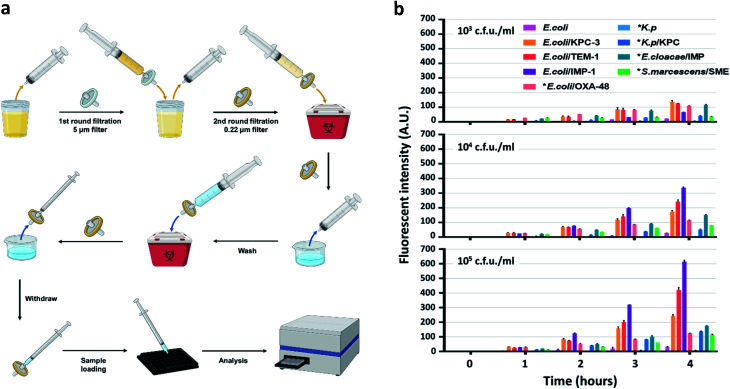
Detection of lactam-resistant bacteria in urine samples. (a) Illustration of the workflow in bacteria isolation and concentration from a urine sample for detection. The sample is first filtered using a 5 μm filter to trap cells and large debris followed by a 0.22 μm filter to trap bacteria. The 0.22 μm filter is washed once before PBS/H_2_O_2_ (1 mM) is directed through the 0.22 μm filter in the opposite direction with a new syringe to recover the concentrated bacteria for detection in a plate reader. (b) Longitudinal monitoring of fluorescence enhancement with CDA and concentrated *E. coli*, *K. pneumoniae*, *K. pneumoniae*/KPC, *E. coli*/TEM-1, *E. coli*/IMP-1, *E. coli*/KPC-3, *E. cloacae*/IMP, *S. marcescens*/SME, and *E. coli*/OXA-48 in the presence of H_2_O_2_ (1 mM). * indicates clinical isolates. The study was duplicated at room temperature with PBS as the buffer. The signal of CDA alone in PBS containing H_2_O_2_ (1 mM) was subtracted and the absolute values were plotted. The working concentration of CDA was 10 μM. Data were collected with excitation at 570 nm and emission at 600 nm. A.U. indicates arbitrary units. Part of the figure was created with http://BioRender.com.

According to the protocol for urinary tract infection (UTI) events updated in January 2020 by the CDC (Centers for Disease Control and Prevention, US), all symptomatic, catheter-associated symptomatic, and asymptomatic bacteremic UTI criteria require a positive urine culture with no more than 2 species of microorganisms, at least one of which is ≥10^5^ cfu mL^−1^.^63^ Some clinical laboratories set a lower threshold (10^3^ to 10^4^ cfu mL^−1^) for reporting UTI under certain circumstances.^64^ Therefore, we prepared samples (∼50 mL each) containing 10^3^, 10^4^, and 10^5^ cfu mL^−1^ of engineered or clinically isolated bacteria confirmed with or without β-lactamases, including *E. coli*, *K. pneumoniae*, *Enterobacter cloacae* (*E. cloacae*) and *Serratia marcescens* (*S. marcescens*).^61^ As shown in Fig. 4b, after 250-fold enrichment, a time dependent increase of the fluorescent signal was observed in all β-lactamase producing bacteria. As negative controls, *E. coli* generated slightly increased signals while those of *K. pneumoniae* were almost undetectable. We utilized 3 times the standard deviation of the mean fluorescence intensity of *E. coli* at different time points to predict the limit of detection: 10^3^ and 10^4^ cfu mL^−1^ of all β-lactamase producing bacteria were detectable within 2 hours of incubation; 10^5^ cfu mL^−1^ could be detected within 1 hour. The fluorescent signals from *E. coli* expressing recombinant β-lactamases were constantly higher than those of clinical isolates, which indicated a more robust β-lactamase expression in these engineered strains.

In summary, we have developed a dual-caged fluorogenic resorufin probe CDA that is stable under physiological conditions with nearly no background but becomes highly fluorescent upon β-lactamase/esterase activation and further oxidation. The cephalosporin moiety provides a wide detection spectrum of β-lactamase expressing bacteria including cephalosporinases and carbapenemases, and thus CDA is suitable for initial screening of broad-spectrum β-lactamase activity, and diagnosis of possibly ESBL-positive and carbapenem resistant pathogens. Modification of the lactam structure for ESBL or carbapenemase specific substrates may further differentiate these mechanisms.^33,65,66^ After a simple two-step filtration, our CDA/H_2_O_2_ assay has been demonstrated to report 10^3^ cfu mL^−1^ of cephalosporin- and carbapenem-resistant bacteria in urine within 2 hours at room temperature. The detection limit may be further improved for example using droplet-based microfluidics.^67^ Zhao *et al.* reported a high-throughput 3D particle counting system for single bacteria detection in blood and more recently for point-of-care diagnosis of UTI in clinics.^68,69^ The integration of these systems may further improve the sensitivity and shorten the assay time. To conclude, dual-caged CDA with cascade activation may facilitate rapid diagnosis of lactam-resistant bacterial pathogens and timely selection of appropriate treatment and prevent further spread of antibiotic resistance.

## Data availability

All the data supporting this article have been included in the main text and the supplementary material.

## Author contributions

J. X., R. M. and J. R. designed and led the study. R. M., M. F. and Y. C. performed the probe synthesis. J. X. performed enzyme purification and probe characterization. F. S., A. M. and N. B. contributed to in culture, isolation, and preparation of the clinical isolates. N. B. contributed to study with clinical isolates. J. X., R. M. and J. R. analyzed all the data and wrote the manuscript with inputs from other authors.

## Conflicts of interest

J. X., R. M. and J. R. are inventors on a U.S. patent application submitted by Leland Junior Stanford University that covers some of the work. All other authors declare that they have no conflict of interest.

## Supplementary Material

SC-012-D1SC01471D-s001
