# Supplementary material for: A dual-caged resorufin probe for rapid screening of infections resistant to lactam antibiotics
Source: Chem Sci. 2021 May 28;12(26):9153–61. doi: 10.1039/d1sc01471d (PMC8261730; doi:10.1039/d1sc01471d)

Electronic Supplementary Material (ESI) for Chemical Science.
This journal is © The Royal Society of Chemistry 2021

Supporting information

A dual-caged resorufin probe for rapid screening of infections resistant to lactam antibiotics

Jinghang Xie[1,‡], Ran Mu[1,‡], Mingxi Fang[1], Yunfeng Cheng[1], Fiona Senchyna[2], Angel Moreno[2], Niaz Banaei[2,3,4], Jianghong Rao[1]*

[1]Departments of Radiology and Chemistry, Molecular Imaging Program at Stanford, Stanford University School of Medicine, Stanford, CA 94305 (USA)

[2]Department of Pathology, Stanford University School of Medicine, Stanford, CA 94305 (USA)

[3]Clinical Microbiology Laboratory, Stanford University Medical Center, Palo Alto, CA 94304 (USA)

[4]Division of Infectious Diseases and Geographic Medicine, Department of Medicine, Stanford University School of Medicine, Stanford, CA 94305 (USA)

‡ Authors J. X. and R. M. contributed equally to this work.

*E-mail: jrao@stanford.edu

Table of contents

Material and methods……………………………………………………………………………………………………..Page 3

1. Chemistry………Page 3

2. Biology……..Page 8

Supplementary figures……………………………………………………………………………………………….Page 11

References……...Page 37

^{1}NMR and ^{13}C NMR spectra………………………………………………………………… ……………………….....Page 38

Materials and Methods

1. Chemistry

1.1 General Information

All chemicals were purchased from commercial sources as specified below. Analytical TLC was performed with 0.25 mm silica gel 60F plates with fluorescent indicator (254 nm). The ^{1}H and ^{13}C NMR spectra were taken on Varian 400 MHz, 500 MHz or 600 MHz magnetic resonance spectrometer. Data for ^{1}H NMR spectra are reported as follows: chemical shifts are reported as δ in units of parts per million (ppm) relative to tetramethylsilane (δ=0, s); multiplicities are reported as follows: s (singlet), d (doublet), t (triplet), q (quartet), dd (doublet of doublets), m (multiplet), or br (broadened); coupling constants are reported as a *J* value in Hertz (Hz); the number of protons (n) for a given resonance is indicated nH, and based on the spectral integration values. HPLC was performed on a Dionex HPLC System (Dionex Corporation) equipped with a GP50 gradient pump and an inline diode array UV-Vis detector. A reversed-phase C18 (Phenomenax, 5 µm, 10 x 250 mm or Dionex, 5 µm, 4.6 x 250 mm) column was used with a MeCN (B) / H_2O (A) gradient mobile phase containing 0.1% trifluoroacetic acid at a flow of 1 or 3 mL/min for the analysis.

1.2 Probe syntheses and characterizations.

Preparation of compound 10*H*-phenoxazine-3,7-diyl diacetate **(2 - DARR)**.

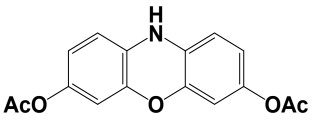

Compound **2 - DARR** was prepared according to a method previously reported.[1] Briefly, step 1: To a 500 mL round-bottom flask and argon sparged, a solution of zinc powder (1.2 g, 20 mmol), resazurin sodium salt (compound **1,** 1 g, 4.0 mmol), and glacial acetic acid (30 mL) were added and stirred vigorously at room temperature for 4 h. After the reacting solvent was evaporated under reduced pressure, a brownish crude product was obtained and used without purifying for next step. Step 2: Under the argon atmosphere, to the same 500 mL round-bottom flask, a stirred solution of the previous crude product in acetone (30 mL) was added N, N-dimethyl-4-aminopyridine (350 mg, 2.1 mmol) in a portion and added dropwise acetic anhydride (1.0 mL, 10.0 mmol). The mixture was stirred

and monitored by TLC for around 6 hours. Zinc powder was removed by Celite filtration, and the filtrate mixture was evaporated under reduced pressure to afford a crude product. Flash column with EtOAc: hexane = 1 : 5 to 1 : 1 gave a yellow crude product in 420 mg.

Preparation of compound 10-(chlorocarbonyl)-10*H*-phenoxazine-3,7-diyl diacetate **(3)**.

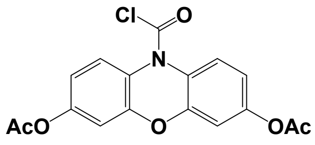

Under argon protection, to an ice-bathed 100mL flask, a solution of 3, 7-diacetoxyphenoxazine (compound **2**) (300 mg, 1.0 mmol) and TEA (0.32 mL, 2.2 mmol) in 10 mL dichloroethane was stirred for 15 min, then it was added dropwisely a solution of triphosgene (1.35 g, 4.5 mmol) in DCE (10 mL) at 0 °C. The reaction mixture was stirred at room temperature under an argon atmosphere for 5 h with HPLC monitoring. Then the mixture was diluted with DCM (60mL) and the resulting mixture was extracted with water (60 mL) and brine (60 mL) three times. The organic layer was extracted and dried with anhydrous Na_2SO_4. A white solid was obtained by silica-gel column chromatography with the eluent of EtOAc: hexane = 1 : 4 to 1 : 1 in 49% yield (181 mg). ^{1}H NMR (400 MHz, $CDCl_3$) δ 7.59 (d, *J* = 8.8 Hz, 2H), 6.97 – 6.95 (m, 2H), 6.92 (dd, *J* = 8.8, 2.5 Hz, 2H), 2.31 (s, 6H); HRMS: Calculated for $C_{17}H_{12}ClNNaO_6$ ($[M+Na]^+$): 384.0245; Found: 384.0241.

Preparation of compound benzhydryl 3-(chloromethyl)-8-oxo-7-(2-phenylacetamido)-5-thia-1-azabicyclo[4.2.0]oct-2-ene-2-carboxylate 5-oxide **(5)**.

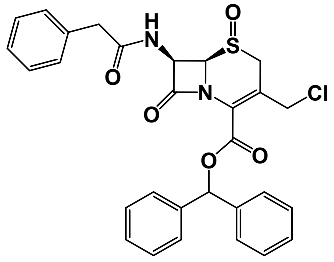

A solution of the cephalosporin chloride precursor (**4**, purchased from Pharmacore, China, 533 mg, 1.0 mmol) in anhydrous DCM was cooled to 0 °C prior to the addition of meta-chloroperoxybenzoic acid (172 mg, 1.0 mmol). The reaction was stirred at 0 °C for 30 min (a white precipitate was formed during this time) and subsequently for another 1 hour at room temperature. Silicon column purification afforded pure compound **5** (550 mg, 98%). ^{1}H NMR (500 MHz, DMSO) δ 8.48 (d, *J* = 8.3 Hz, 1H), 7.53 (d, *J* = 7.2 Hz, 2H), 7.44 (d, *J* = 7.2 Hz, 2H), 7.36 (td, *J* = 7.5, 4.4 Hz, 4H), 7.30 (m, 6H), 7.27 – 7.21 (m, 1H), 6.99 (s, 1H), 5.93 (dd, *J* = 8.3, 4.9 Hz, 1H), 5.03 – 4.93 (m, 1H), 4.62 (d, *J* = 11.6 Hz, 1H), 4.46 (d, *J* = 11.4 Hz, 1H), 3.95 (d, *J* = 18.3 Hz, 1H), 3.79 – 3.61 (m, 2H), 3.56 (d, *J* = 14.0 Hz, 1H); ^{13}C NMR (126 MHz, DMSO) δ 171.56, 165.03, 160.05, 140.07, 136.26, 129.56, 129.04, 128.90, 128.76, 128.42, 127.34, 127.02, 126.96, 125.18, 122.16, 79.64, 67.17, 58.53, 46.58, 44.23, 41.85; HRMS: Calculated for $C_{29}H_{25}ClN_2O_5S$ ($[M+Na]^+$): 571.1065; Found: 571.1060.

Preparation of compound benzhydryl 3-(((4-(hydroxymethyl)phenyl)thio)methyl)-8-oxo-7-(2-phenylacetamido)-5-thia-1-azabicyclo[4.2.0]octane-2-carboxylate 5-oxide (**6**)

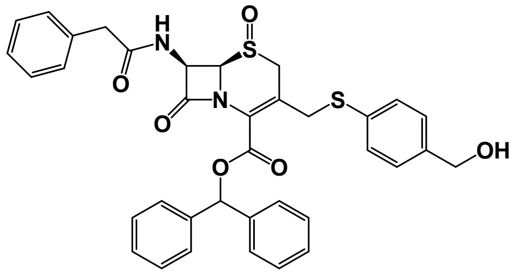

After sonication, a mixture of **compound 5** (440 mg, 0.8 mmol) and potassium carbonate (55.2 mg, 1.2 mmol) in anhydrous acetonitrile (10 mL) was stirred at room temperature for 30 min, then (4-mercaptophenyl)methanol (110.4 mg, 0.8 mmol) was added and the resulting mixture was stirred at room temperature for overnight. EtOAc (100 mL) and H_2O (20 mL) was added to the reaction solution, and the separated organic layer was further washed with H_2O (20 mL) twice and saturated brine (20 mL). After removal of the solvent under Rota-Vap, the crude product was purified by flash chromatography with a silica gel column (v/v 0-8% MeOH in DCM) to afford compound **6** (235 mg, 45%). ^{1}H NMR (500 MHz, DMSO) δ 8.42 (d, *J* = 8.4 Hz, 1H), 7.50 (d, *J* = 7.4 Hz, 2H), 7.36 (s, 3H), 7.32 (d, *J* = 8.4 Hz, 4H), 7.31 – 7.26 (m, 8H), 7.17 (d, *J* = 2.0 Hz, 4H), 6.80 (s, 1H), 5.85 (dd, *J* = 8.3,

4.8 Hz, 1H), 4.90 (d, J = 3.5 Hz, 1H), 4.35 (s, 2H), 4.30 (d, J = 13.4 Hz, 1H), 3.95 (d, J = 18.4 Hz, 1H), 3.80 (d, J = 18.8 Hz, 1H), 3.76 (d, J = 13.4 Hz, 1H), 3.68 (d, J = 14.0 Hz, 1H), 3.56 (d, J = 14.0 Hz, 1H); ^{13}C NMR (126 MHz, DMSO) δ 171.48, 164.77, 160.26, 142.26, 140.22, 140.13, 136.16, 132.11, 132.00, 131.39, 129.57, 128.99, 128.84, 128.75, 128.30, 127.55, 127.35, 127.01, 126.90, 124.26, 123.77, 79.30, 62.69, 58.28, 47.46, 41.92; HRMS: Calculated for $C_{36}H_{35}N_2O_6S_2$ ($[M+H]^+$): 655.1920; Found: 655.1931.

Preparation of compound 10-(((4-(((2-((benzhydryloxy)carbonyl)-5-oxido-8-oxo-7-(2-phenylacetamido)-5-thia-1-azabicyclo[4.2.0]oct-2-en-3-yl)methyl)thio)benzyl)oxy)carbonyl)-10*H*-phenoxazine-3,7-diyl diacetate (**7**)

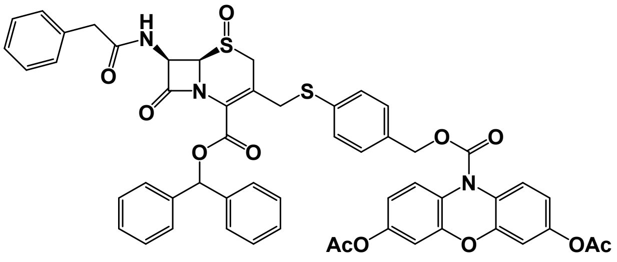

Under an argon atmosphere, to a solution of K_2CO_3 (31 mg, 0.23 mmol) and N,N-dimethyl-4-aminopyridine (3 mg, 0.04 mmol) in DCE (5 mL) was in dropwise added a solution of compound **6** (105.9 mg, 0.162 mmol) in DCE (5 mL) at 0 °C. After 30 min, compound **3** (62 mg, 0.17 mmol) dissolved in DCE (10 mL) was added dropwise and the resulting mixture was stirred at room temperature for another 5 hours, monitored by TLC. Then the mixture was diluted with DCM (50 mL) and extracted with water (60 mL) and brine (60 mL) three times. The organic layer was dried with Na_2SO_4, concentrated, and flash silica-gel column (5% methanol in DCM) gave crude compound **7** which was used in the next step without further purification.

Preparation of compound 10-(((4-(((2-((benzhydryloxy)carbonyl)-8-oxo-7-(2-phenylacetamido)-5-thia-1-azabicyclo[4.2.0]oct-2-en-3-yl)methyl)thio)benzyl)oxy)carbonyl)-10H-phenoxazine-3,7-diyl diacetate (**8**)

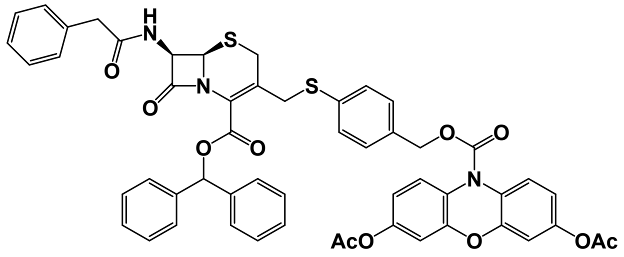

The reduction of compound **7** was performed according to the method previously reported.[2] Under argon protection, to a mixture of **7** and NaI (30.1 mg, 0.2 mmol) in anhydrous acetone (10 mL) at ice bath was added dropwise trifluoroacetic anhydride (TFAA) (34 µL, 0.25 mmol). The resulting mixture was stirred at 0 °C for one hour. After removal of the solvent under Rota-Vap, the residue was dissolved in $NaHCO_3$ (aq.) (5 mL) and extracted with ethyl acetate (5 mL x 3). The titled product was purified by a silica gel column (v/v 0-8% MeOH in DCM) as solid (38 mg) at an overall yield of 25% from compound **6**. ^{1}H NMR (500 MHz, DMSO) δ 9.17 (s, 1H), 7.59 (d, J = 8.9 Hz, 2H), 7.33-7.25 (m, 19H), 7.04 (d, J = 2.6 Hz, 2H), 6.95 (d, J = 2.5 Hz, 2H), 6.85 (s, 1H), 6.57 (s, 1H), 5.47 (s, 1H), 5.34 (s, 1H), 5.24 (s, 2H), 5.12 (d, J = 3.9 Hz, 1H), 3.94 (q, 1H), 3.63 (s, 1H), 3.51 (d, J = 4.5 Hz, 2H), 2.25 (s, 6H); ^{13}C NMR (126 MHz, $CDCl_3$) δ 171.53, 169.13, 166.25, 164.17, 153.01, 150.41, 148.61, 138.85, 134.69, 134.64, 133.62, 132.32, 131.83, 131.49, 129.86, 129.42, 129.17, 129.02, 128.91, 128.68, 128.47, 128.13, 127.57, 127.19, 126.78, 126.40, 125.81, 125.78, 125.74, 125.42, 125.10, 119.63, 118.83, 117.06, 116.31, 110.98, 110.21, 79.57, 68.41, 67.96, 67.29, 60.43, 60.02, 53.93, 53.12, 52.67, 50.72, 50.33, 49.64, 43.04, 40.34, 21.14, 21.00; HRMS: Calculated for $C_{53}H_{43}N_3NaO_{11}S_2$ ([M+Na]$^+$): 984.2231; Found: 984.2221.

Preparation of compound 3-(((4-(((3,7-diacetoxy-10H-phenoxazine-10-carbonyl)oxy)methyl)phenyl)thio)methyl)-8-oxo-7-(2-phenylacetamido)-5-thia-1-azabicyclo[4.2.0]oct-2-ene-2-carboxylic acid (**CDA**)

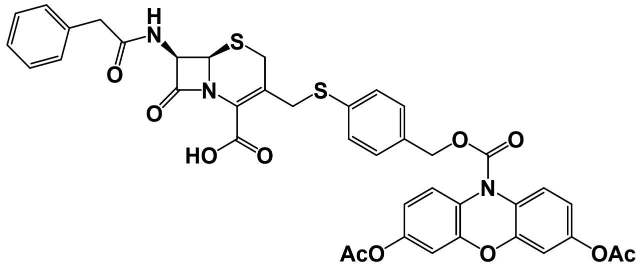

A pre-cold mixed solution of DCM (10.5 mL), trifluoroacetic acid (TFA, 3 mL) and triisopropylsilane (TIPS, 1.5 mL) was added to a flask pre-filled with compound **8** (25 mg, 0.026 mmol). The resulting mixture was then stirred at room temperature for 1 hour. After condensation under reduced vacuum, the crude was purified by HPLC (0.1% acetonitrile from 40% to 100%) to afford probe **CDA** (10 mg, 48%). ^{1}H NMR (600 MHz, DMSO) δ 9.14 (d, J = 7.7 Hz, 1H), 7.60 (d, J = 8.9 Hz, 2H), 7.39 – 7.34 (m, 4H), 7.28 (t, J = 7.4 Hz, 2H), 7.24 (d, J = 6.6 Hz, 2H), 7.21 (d, J = 7.2 Hz, 1H), 7.04 (d, J = 2.6 Hz, 2H), 6.96 (dd, J = 8.8, 2.6 Hz, 2H), 6.50 (s, 2H), 5.41 (dd, J = 7.7, 3.9 Hz, 1H), 5.24 (s, 2H), 5.13 (d, J = 3.8 Hz, 1H), 5.07 (d, J = 1.7 Hz, 1H), 4.00 (d, J = 14.3 Hz, 1H), 3.77 (d, J = 14.2 Hz, 1H), 3.50 (d, J = 4.8 Hz, 2H), 2.25 (s, 6H) ; ^{13}C NMR (126 MHz, $CDCl_3$) δ 171.56, 169.72, 168.58, 164.20, 153.04, 150.40, 148.52, 134.95, 134.36, 133.58, 131.42, 129.49, 129.10, 128.31, 127.69, 125.76, 125.35, 119.67, 118.48, 116.96, 110.50, 68.01, 60.16, 53.14, 49.87, 43.10, 40.05, 29.97, 29.72, 21.14; HRMS: Calculated for $C_{40}H_{34}N_3O_{11}S_2$ ($[M+H]^+$): 796.1629; Found: 796.1619.

2. Biology

2.1 General information

TALON metal affinity resin for affinity purification was purchase from Clontech (Mountain view, CA). Amplex red reagent (A12222), SimpleBlue SafeStain protein gel staining reagent, and fluorescein diacetate were purchased from Invitrogen (Carlsbad, CA). Hydrogen peroxide, 30% solution, was purchased from Thermo Fisher Scientific (Waltham, MA). UV-vis spectra were recorded on an Agilent spectrophotometer. Fluorescence Spectra were collected by a SpectraMax iD3 multimode microplate reader (Molecular Device, San Jose, CA). Kinetic experiments were conducted in a Safire microplate

reader (TECAN, research triangle park, NC). Esterase from *Rhizopus oryzae* and propidium iodide were purchased from SIGMA (St. Louis, MO).

2.2. Expression, purification and SDS-PAGE analysis of β-lactamases

The clone of TEM-1 Bla, BlaC, OXA48, AmpC, IMP-1 and KPC-3 was described previously.[3] The general expression and purification procedures were described previously.[4] Briefly, a single colony of *E. coli* (BL21 or TOP10) containing pBAD-TEV(Cys)-TEM1, pET28b-BlaC, pBAD-IMP1-6xHis, pBAD-AmpC-6xHis, pBAD-KPC3-6xHis and pBAD-OXA48-6xHis was inoculated into 100 mL of Lysogeny broth (LB broth) with 100 µg/mL ampicillin, followed by incubation at 37 °C, 205 rpm overnight. The overnight culture was added into 500 mL of fresh LB broth with antibiotic and 0.2% arabinose. After incubation for up to 6 h at 30 °C, 205 rpm, bacteria were harvested, washed, and the pellet was frozen at -80 °C. The pellets were later resuspended and lysed in Novagen Bugbuster protein extraction reagent (EMD Millipore, Burlington, MA). Proteinase inhibitor cocktail (cOmplete, mini, EDTA-free, Roche) was added to the bacterial lysate before two rounds of metal-affinity purification using TALON metal affinity resin. The affinity purified fraction was eluted using lysis buffer containing up to 500 mM imidazole. Buffer was changed to PBS (supplemented with 10% glycerol) by centrifugation with centrifugal filter units (EMD Millipore, Burlington, MA) at regenerated size 30K. Protein samples were denatured in LDS loading buffer (Life Technologies, Carlsbad, CA) with a heating block and analyzed by SDS-PAGE (NuPAGE, Life Technologies, Carlsbad, CA). The gels were stained by SimpleBlue SafeStain protein gel staining reagent. BlaC was expressed and purified as previously described.[5]

2.3. Bacteria growth and assay

E. coli (BL21 or TOP10) transformed to express TEM-1 Bla, IMP-1, and KPC3 were grown in LB medium and induced with 0.2% arabinose for 6-8 h at 30 °C, 205 rpm. Colony forming units per milliliter (c.f.u./mL) were determined by measuring the UV absorbance at OD_{600}.

For CDA incubation, 10 µM working solution was prepared freshly by diluting stock solution (1 mM in pure DMSO) in PBS (pH 7.05). A light-safe tube or foil wrap was used to prevent photobleaching.

Clinically isolated *K. pneumoniae*, *K. pneumoniae* expressing KPC, *E. cloacae* expressing IMP, *E. coli* expressing OXA-48 and *S. marcescens* expressing SME were cultured in BD Columbia agar plate containing 5% sheep blood. Resistant bacteria colonies near meropenem discs were further inoculated in nutrient broth to culture overnight before use.

3. Statistical Analysis

GraphPad Prism 7 was used for plotting and statistical analysis. The statistical difference was determined by performing two-way ANOVA (Figure 3d) followed by Bonferroni's multiple comparison test to determine the statistical significance with 95% confidence intervals with $^{*}p < 0.0332$; $^{**}p < 0.0021$, $^{***}p < 0.0002$, $^{****}p < 0.0001$, ns: not significant.

Supplementary figures

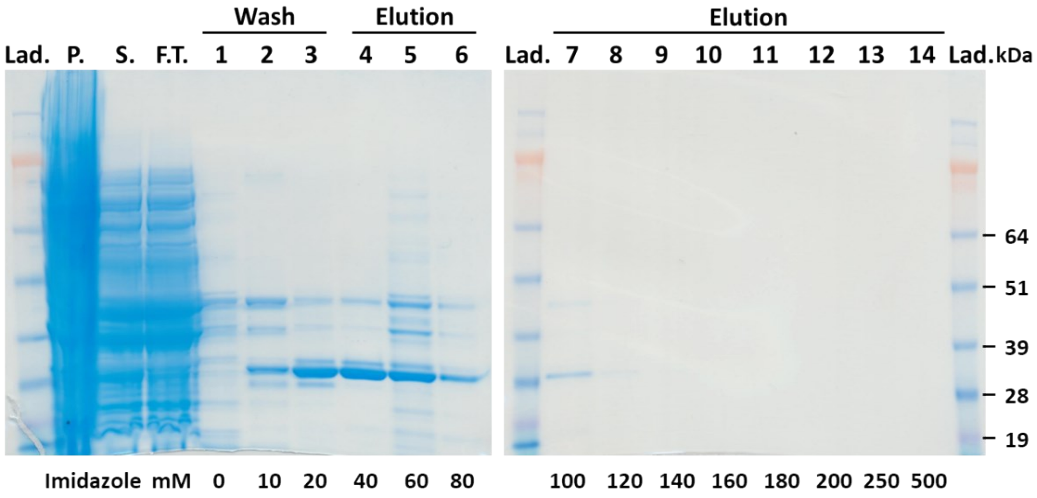

Figure S1. SDS-PAGE analysis of purified TEM-1 Bla. Lad: SeeBlue Plus 2 pre-stained protein standard; P: pellet of lysate; S: supernatant of lysate; F.T.: flow through fraction of supernatant from TALON resin column; Wash by HEPES buffer; Lanes 1-3: wash by 0, 10, or 20 mM imidazole; Lanes 4-14: elution from the TALON resin column with 40, 60, 80, 100, 120, 140, 160, 180, 200, 250 or 500 mM imidazole. The gel was stained by SimpleBlue staining reagent.

a

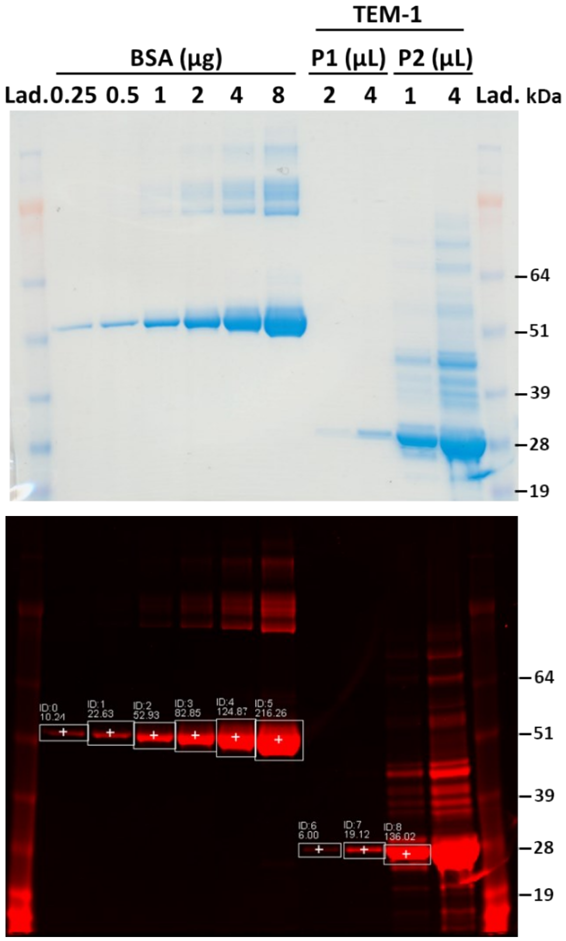

b

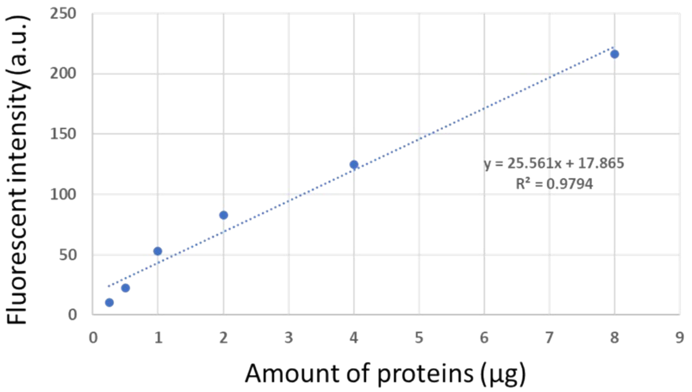

Figure S2. Quantification of purified TEM-1 Bla. **a)** SDS-PAGE analysis of purified TEM-1 Bla and BSA as standard. Lad: SeeBlue Plus 2 pre-stained protein standard. The gel was stained by SimpleBlue staining reagent. Upper panel: scan under bright field; lower panel: scan at 800 nm by a LI-COR odyssey scanner and near-infrared intensity defined by regions of interest (ROIs). **b)** Standard curve generated by plotting near-infrared intensity and the amount of BSA loaded to normalize the concentration of TEM-1 Bla.

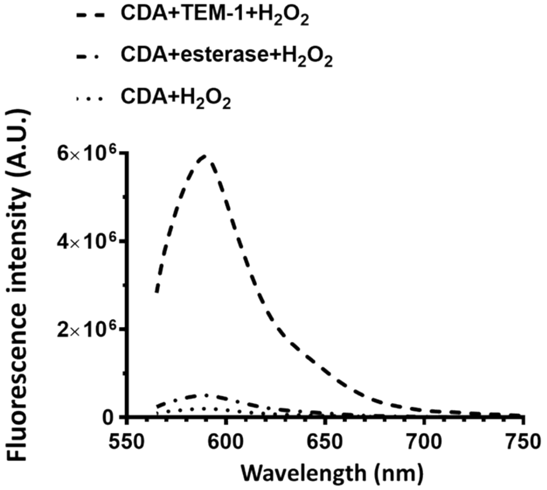

Figure S3. Fluorescent emission spectra of CDA treated with esterase (1 μg/mL), TEM-1 Bla (100 nM) and H_2O_2 (1 mM) at room temperature for 8 hours. A.U. indicates arbitrary units.

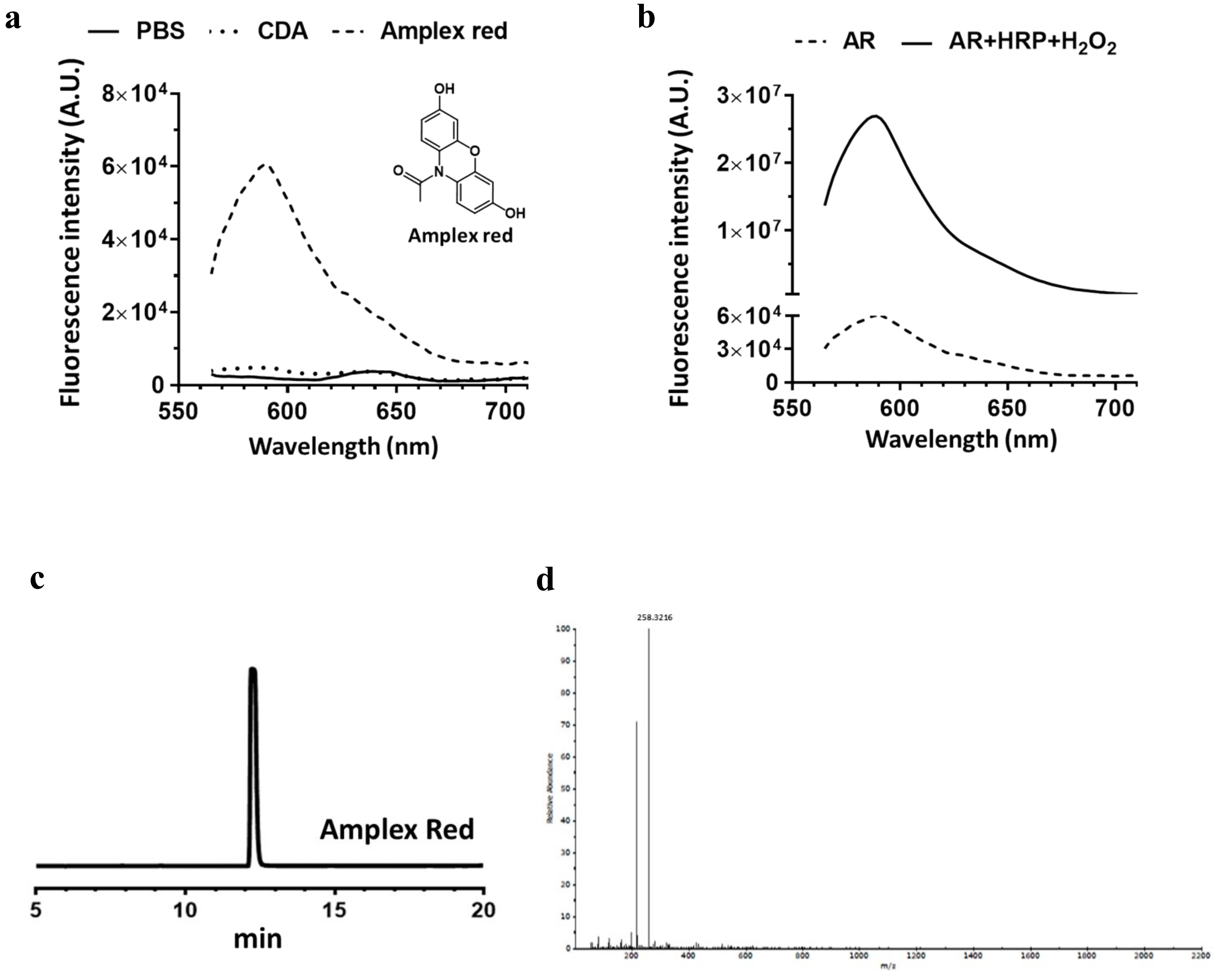

Figure S4. Characterization of CDA and Amplex red (AR). **a)** Fluorescent emission spectra of PBS, CDA, and AR (10 μM probes in PBS; excitation, 525 nm). **b)** Fluorescent emission spectra of AR and AR treated with HRP and H_2O_2 at room temperature for 5 min (10 μM AR in PBS, HRP 1 unit/mL, H_2O_2 1 μM) (excitation, 525 nm). A.U. indicates arbitrary units. **c)** HPLC traces of Amplex red. **d)** Mass spectra of Amplex red (calculated M.W. 257.24) in positive electrospray ionization modes.

a

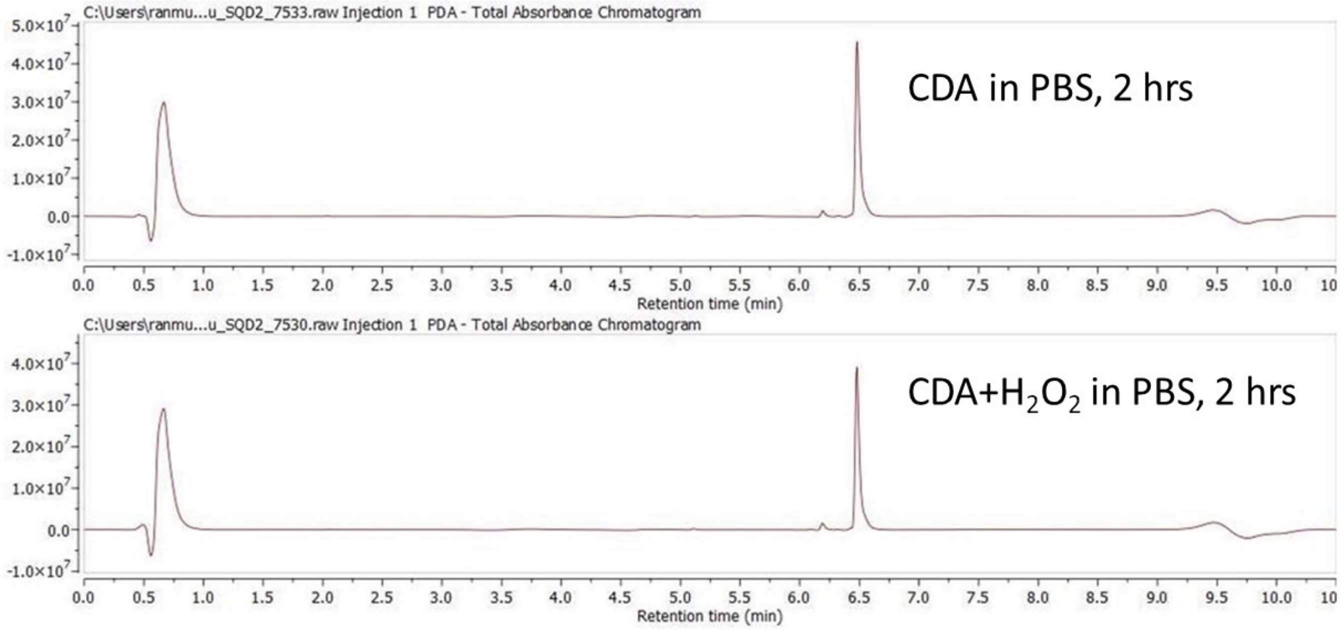

b

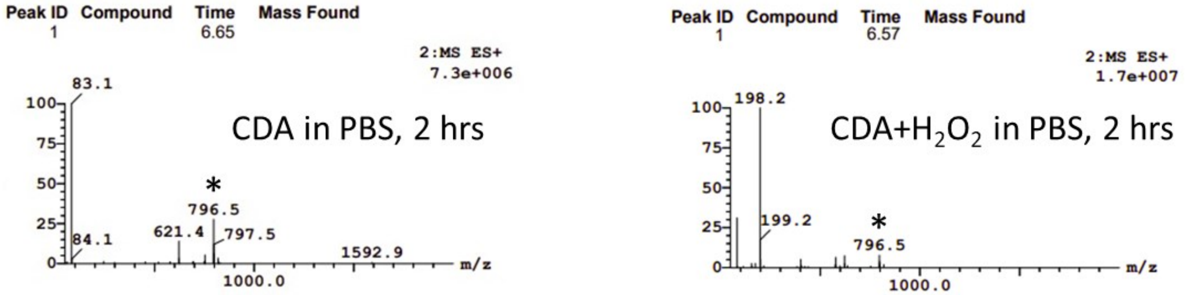

Figure S5. LC-MS analysis of CDA and CDA in 1 mM H_2O_2 at room temperature for 2 hours. **a)** HPLC traces of CDA with or without H_2O_2. **b)** Mass spectra of CDA with or without H_2O_2 in positive electrospray ionization modes. Peak * indicates CDA (calculated M.W. 795.83).

a

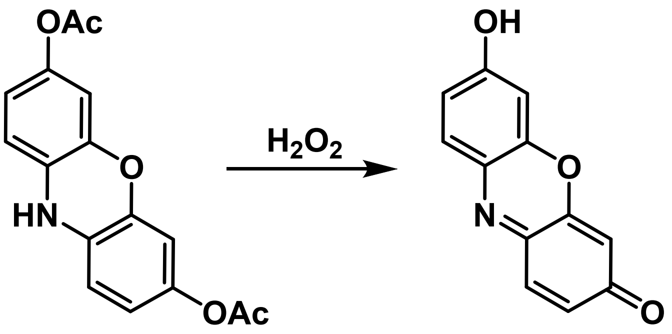

b

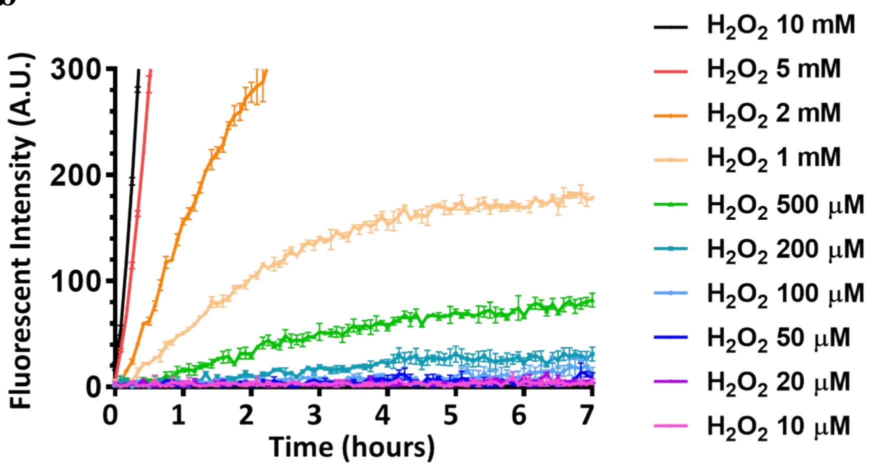

Figure S6. Stability of DARR in H_2O_2**. a)** Structure of DARR and its oxidation by H_2O_2 into fluorescent resorufin. **b)** Stability test of DARR (10 μM) in different concentrations of H_2O_2 at room temperature. Data were collected on a Tecan Safire microplate reader with excitation at 570 nm and emission at 600 nm. A.U. indicates arbitrary units.

a

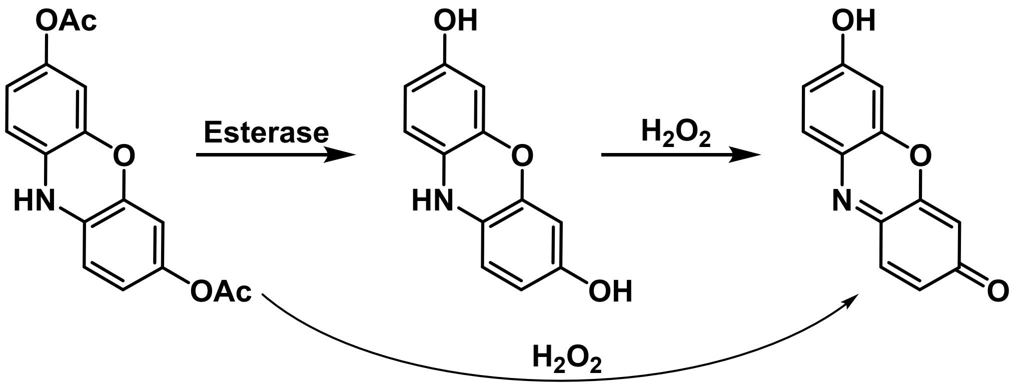

b

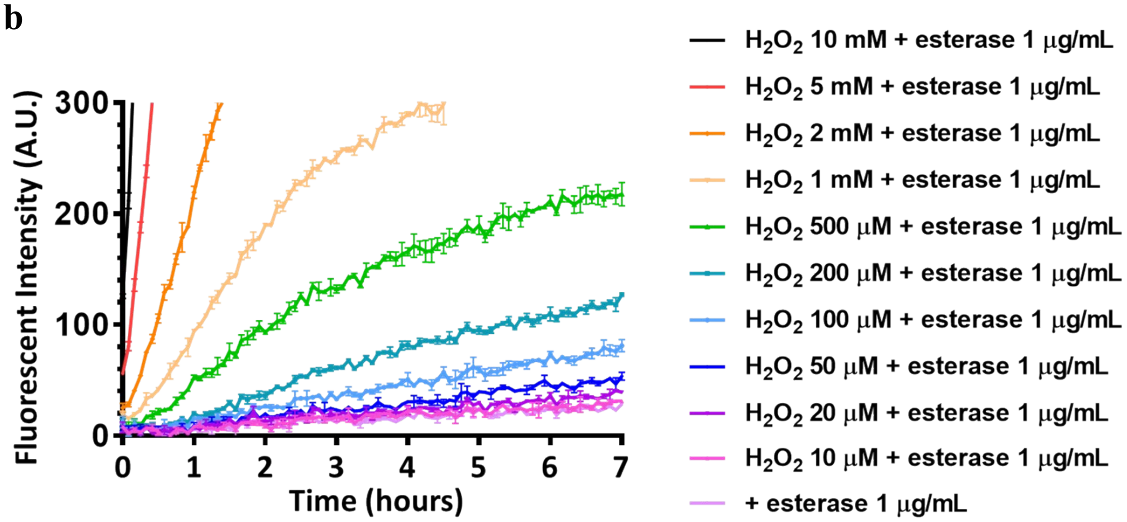

Figure S7. Esterase sensitizes DARR in response to H_2O_2. **a)** Structure of DARR and its hydrolysis by esterase and oxidation into fluorescent resorufin by H_2O_2. **b)** Fluorescence enhancement of DARR treated with esterase (1 μg/mL) and different concentrations of H_2O_2 at room temperature. Data were collected on a Tecan Safire microplate reader with excitation at 570 nm and emission at 600 nm. A.U. indicates arbitrary units.

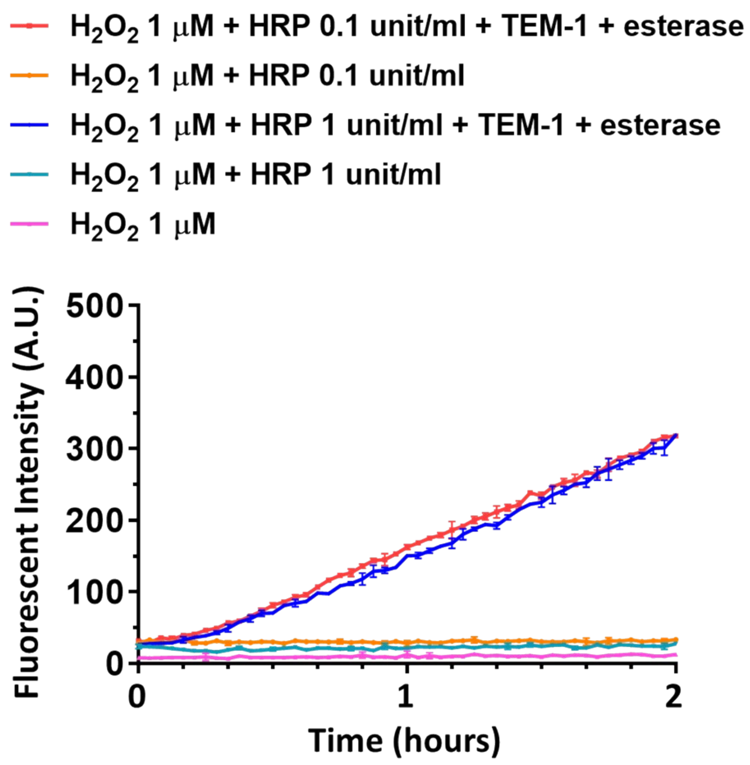

Figure S8. Fluorescence change of CDA (10 μM) in the presence of esterase (1 μg/mL), TEM-1 Bla (100 nM), H_2O_2 (1 μM) and/or HRP (0.1 or 1 unit/mL) at room temperature in PBS (pH=7.05). Data were collected on a Tecan Safire microplate reader with excitation at 570 nm and emission at 600 nm. A.U. indicates arbitrary unit.

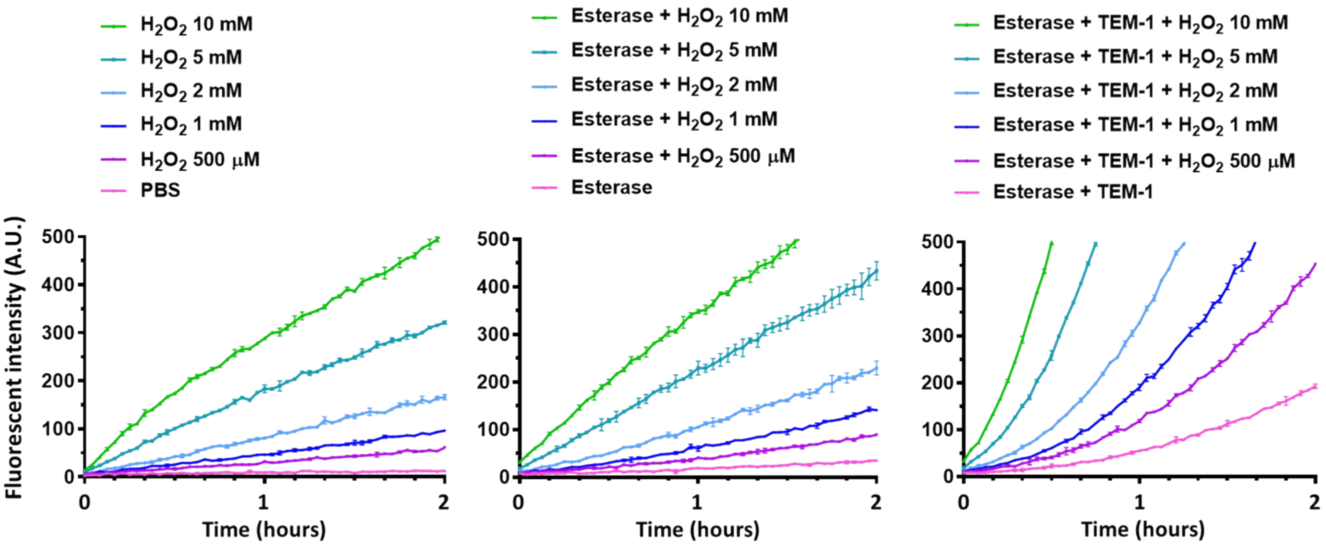

Figure S9. Response of CDA (10 μM) to different concentration of H_2O_2 in the presence of recombinant TEM-1 Bla (100 nM) and esterase (1 μg/mL) at room temperature in PBS (pH=7.05). Data were collected on a Tecan Safire microplate reader with excitation at 570 nm and emission at 600 nm. A.U. indicates arbitrary unit.

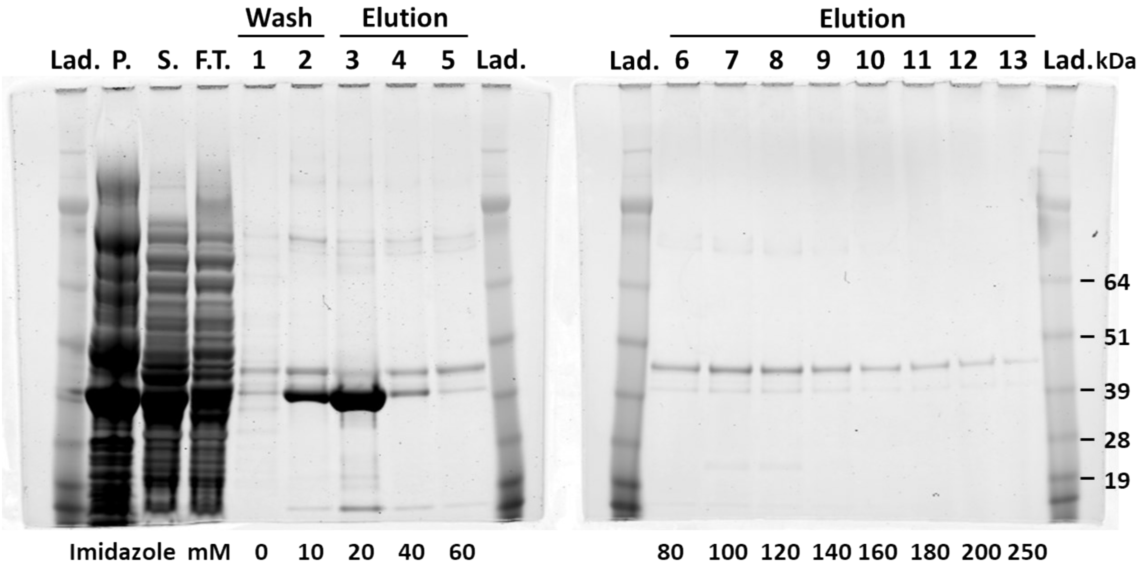

Figure S10. SDS-PAGE analysis of purified AmpC. Lad: SeeBlue Plus 2 pre-stained protein standard; P: pellet of lysate; S: supernatant of lysate; F.T.: flow through fraction of supernatant from TALON resin column; Wash by HEPES buffer-1 and HEPES buffer containing 10 mM imidazole-2; Lanes 3-13: elution from the TALON resin column with 20, 40, 60, 80, 100, 120, 140, 160, 180, 200 or 250 mM imidazole. The gel was stained by SimpleBlue staining reagent and scanned with a LI-COR Odyssey.

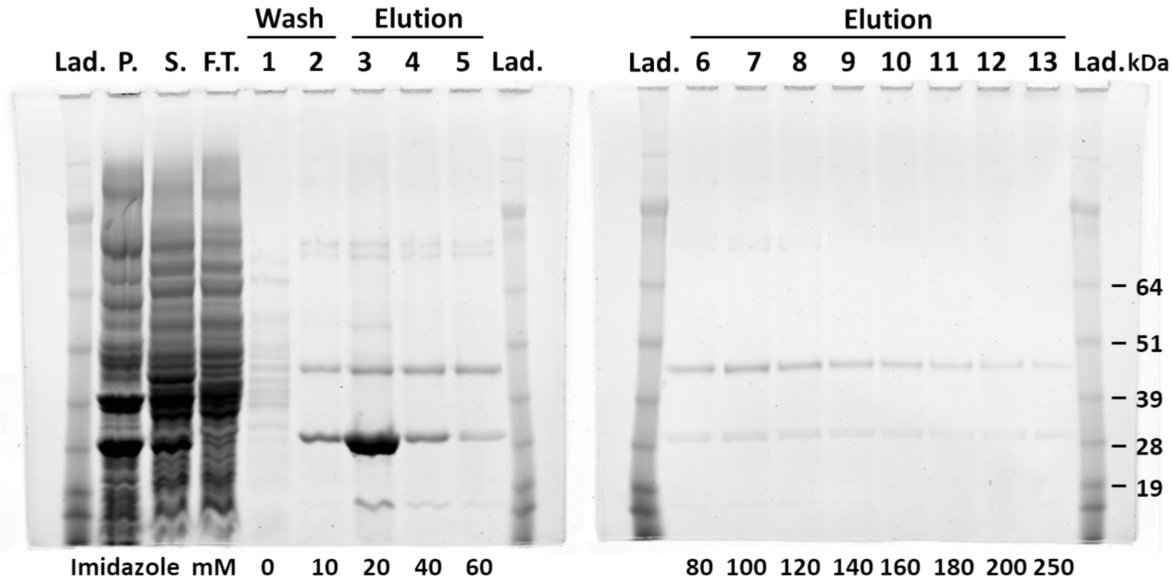

Figure S11. SDS-PAGE analysis of purified OXA-48. Lad: SeeBlue Plus 2 pre-stained protein standard; P: pellet of lysate; S: supernatant of lysate; F.T.: flow through fraction of supernatant from TALON resin column; Wash by HEPES buffer-1 and HEPES buffer containing 10 mM imidazole-2; Lanes 3-13: elution from the TALON resin column with 20, 40, 60, 80, 100, 120, 140, 160, 180, 200 or 250 mM imidazole. The gel was stained by SimpleBlue staining reagent and scanned with a [illegible]

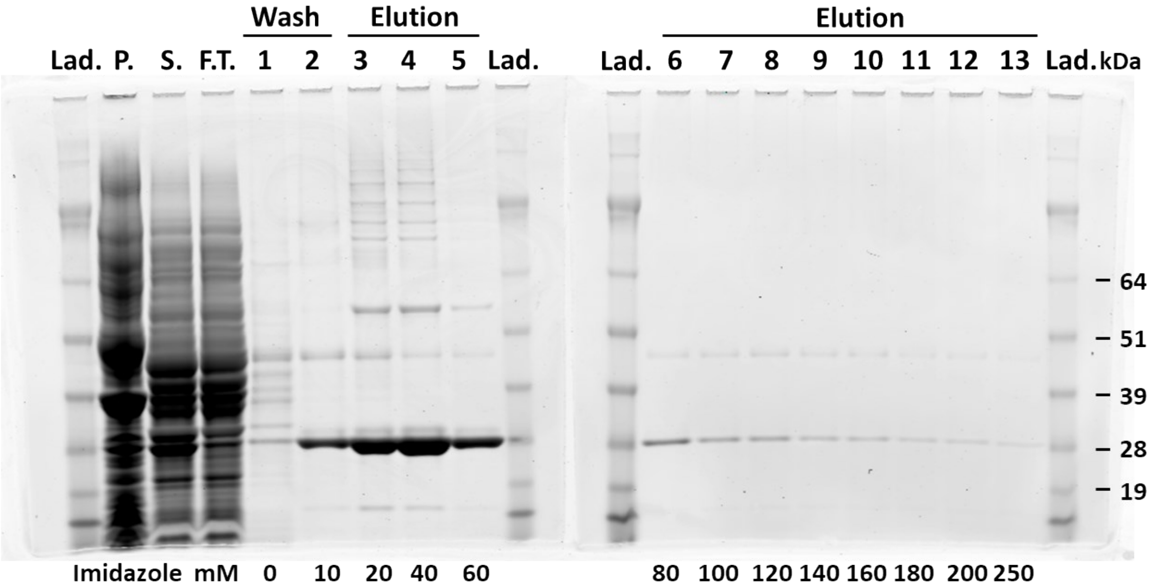

Figure S12. SDS-PAGE analysis of purified KPC-3. Lad: SeeBlue Plus 2 pre-stained protein standard; P: pellet of lysate; S: supernatant of lysate; F.T.: flow through fraction of supernatant from TALON resin column; Wash by HEPES buffer-1 and HEPES buffer containing 10 mM imidazole-2; Lanes 3-13: elution from the TALON resin column with 20, 40, 60, 80, 100, 120, 140, 160, 180, 200 or 250 mM imidazole. The gel was stained by SimpleBlue staining reagent and scanned with a LI-COR Odyssey.

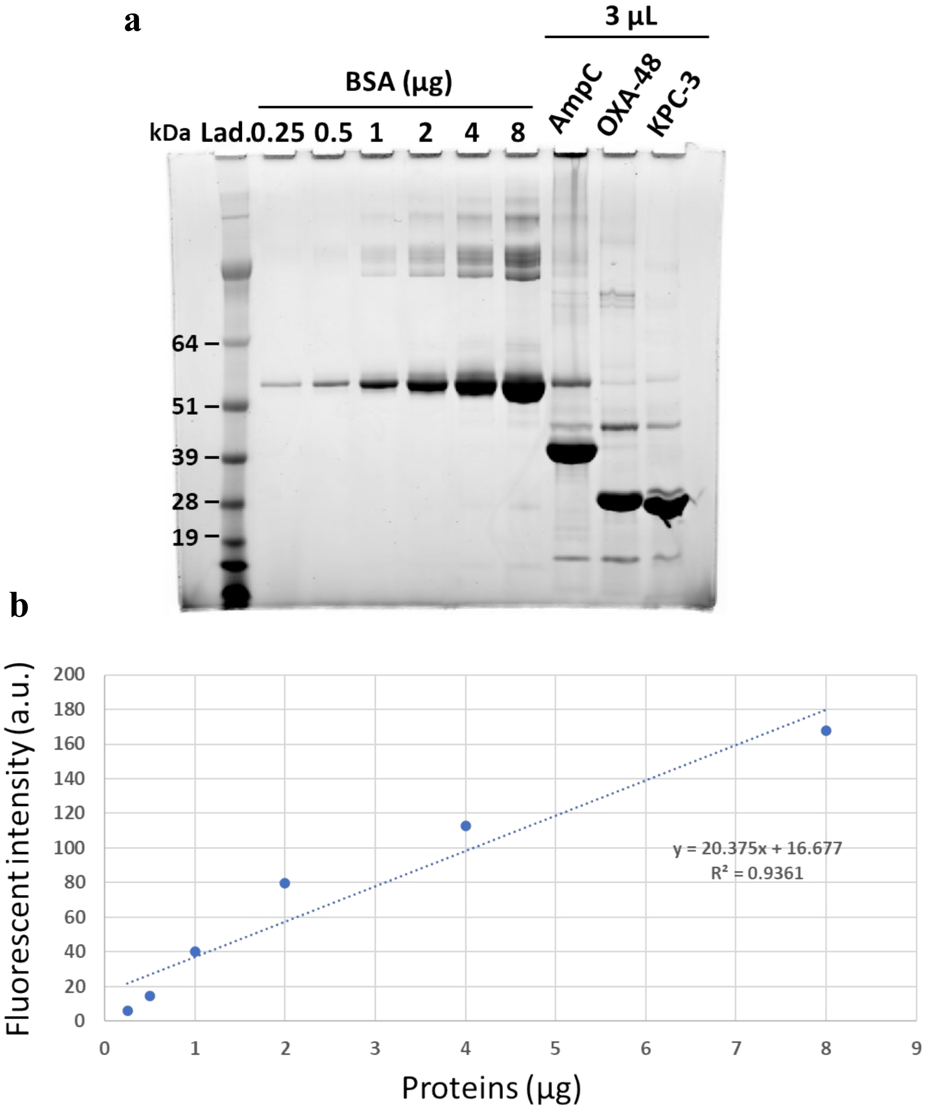

Figure S13. Quantification of purified AmpC, OXA-48 and KPC-3. **a)** SDS-PAGE analysis of purified AmpC, OXA-48, KPC-3 and BSA as standard. Lad: SeeBlue Plus 2 pre-stained protein standard. The gel was stained by SimpleBlue staining reagent. The gel was scanned at 800 nm by a LI-COR odyssey scanner and near-infrared intensity was defined by regions of interest (ROIs). **b)** Standard curve generated by plotting near-infrared intensity and the amount of BSA loaded to normalize the concentration of β-lactamases.

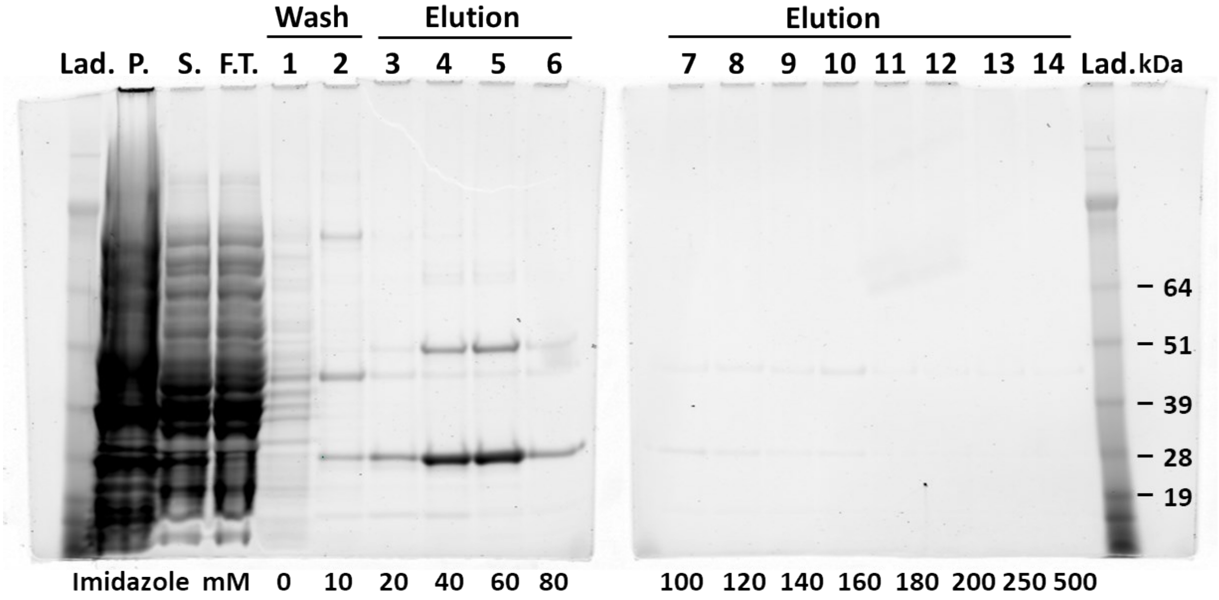

Figure S14. SDS-PAGE analysis of purified IMP-1. Lad: SeeBlue Plus 2 pre-stained protein standard; P: pellet of lysate; S: supernatant of lysate; F.T.: flow through fraction of supernatant from TALON resin column; Wash by HEPES buffer-1 and HEPES buffer containing 10 mM imidazole-2; Lanes 3-14: elution from the TALON resin column with 20, 40, 60, 80, 100, 120, 140, 160, 180, 200, 250 and 500 mM imidazole. The gel was stained by SimpleBlue staining reagent and scanned with a LI-COR [illegible]

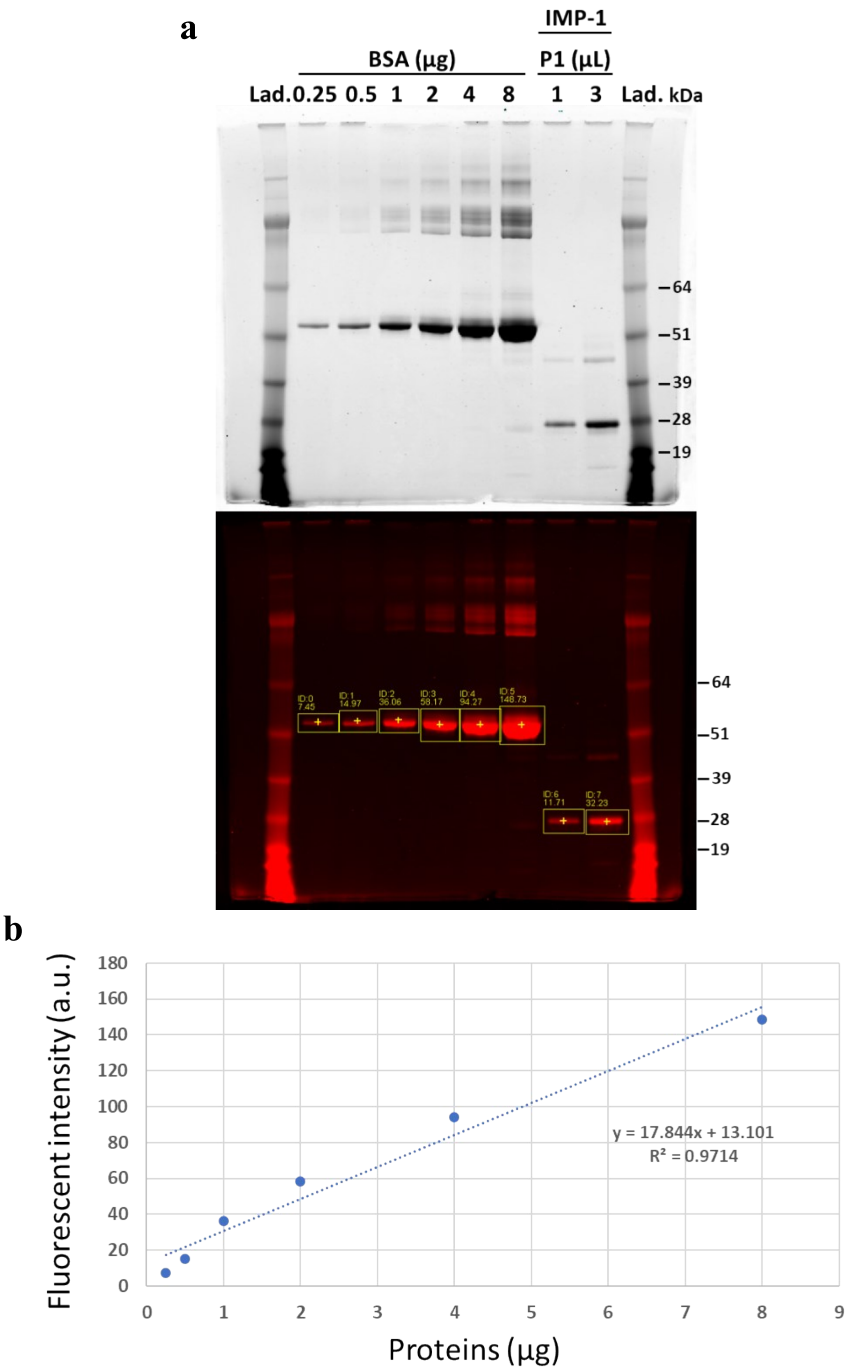

Figure S15. Quantification of purified IMP-1. **a)** SDS-PAGE analysis of purified IMP-1 and BSA as standard. Lad: SeeBlue Plus 2 pre-stained protein standard. The gel was stained by SimpleBlue staining reagent. Upper panel: scan under bright field; lower panel: scan at 800 nm by a LI-COR odyssey scanner and near-infrared intensity defined by regions of interest (ROIs). **b)** Standard curve generated by plotting near-infrared intensity and the amount of BSA loaded to normalize the concentration of IMP-1.

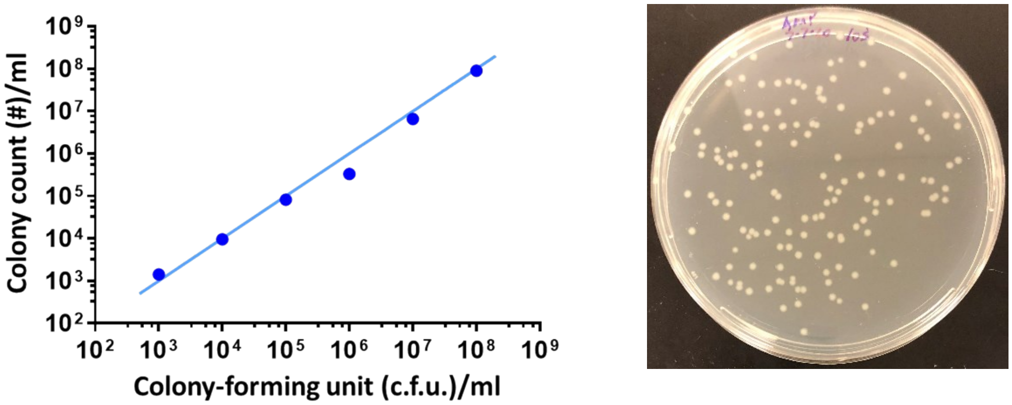

Figure S16. Calibration of the optical density (OD) and *E. coli* colony-forming unit (c.f.u.). Left: the correlation of c.f.u./mL calculated from OD_{600} absorbance to colony count/mL on agar plates. Right: a representative agar plate with around 100 colonies from 100 µL of 10^3 c.f.u./mL *E. coli* suspension determined by measuring its absorbance at OD_{600}.

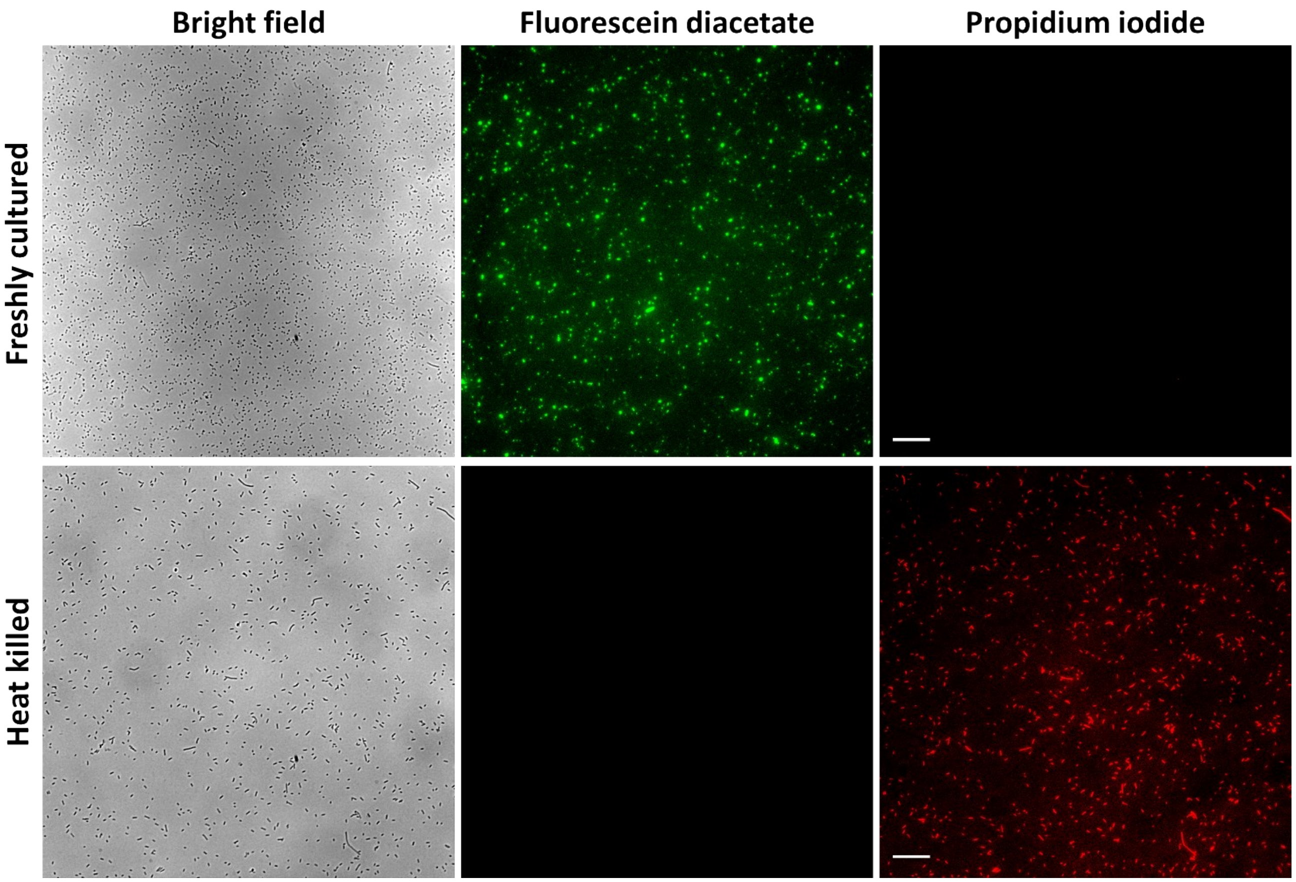

Figure S17. Freshly cultured and heat killed (120 °C autoclave, 15 min) *E. coli* stained with fluorescein diacetate (Ex490/Em520) and propidium iodide (Ex535/Em617). Scale bars represent 25 μm.

a

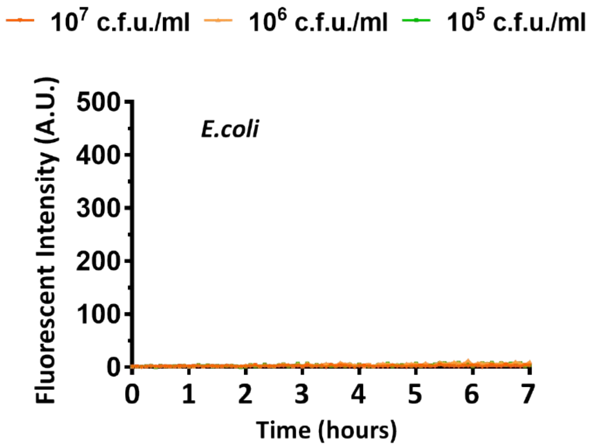

b

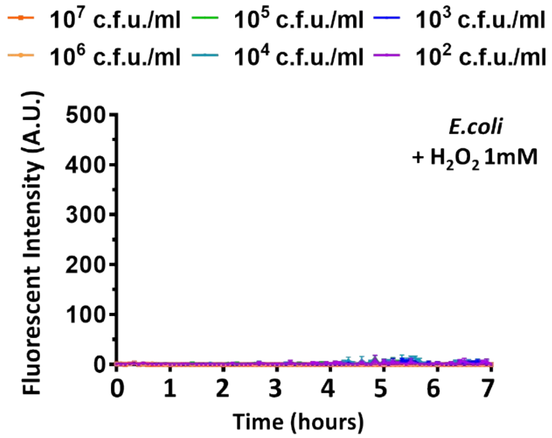

Figure S18. Fluorescence enhancement of CDA with different concentrations of *E. coli* with or without H_2O_2. The study was duplicated at room temperature with PBS (pH=7.05) as the buffer. The working concentration of CDA was 10 µM. The signal of CDA in PBS (a) or H_2O_2 (b) was subtracted and the absolute values were plotted. Data were collected on a Tecan Safire microplate reader with excitation at 570 nm and emission at 600 nm. A.U. indicates arbitrary unit.

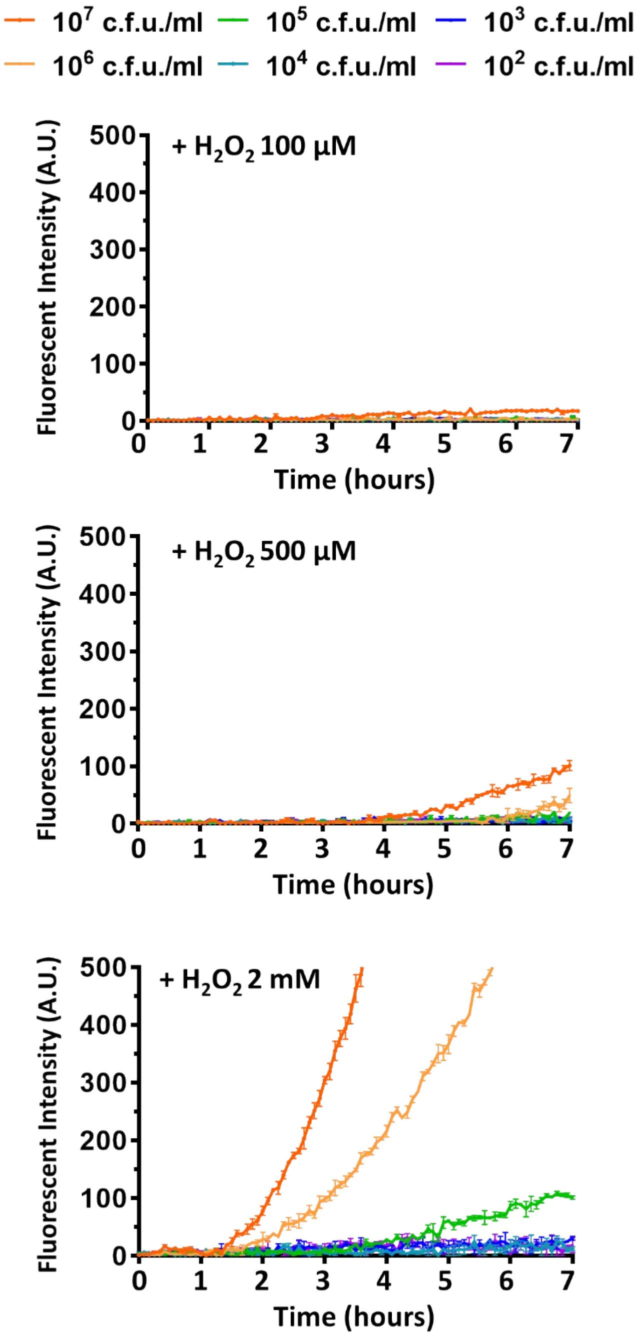

Figure S19. Fluorescence change of CDA with different concentrations of *E. coli* expressing TEM-1 Bla in the presence of H_2O_2 (100 µM, 500 µM, 2 mM). The signal of CDA in corresponding concentration of H_2O_2 were subtracted and the absolute values were plotted. All the studies were performed at room temperature in PBS (pH=7.05). The working concentration of CDA was 10 µM. Data were collected on a Tecan Safire microplate reader with excitation at 570 nm and emission at 600 nm. A.U. indicates arbitrary unit.

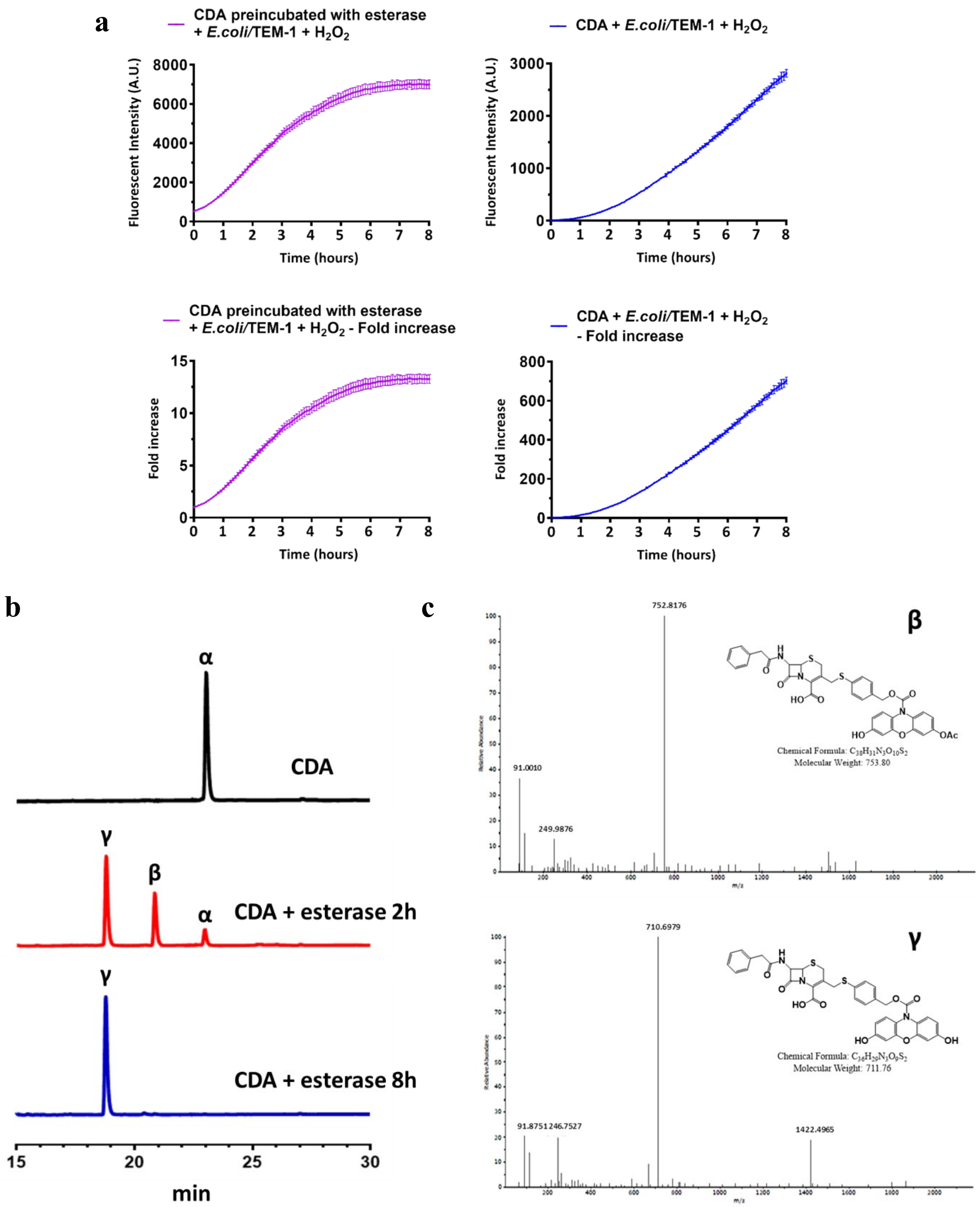

Figure S20. Characterization of the function of esters in **CDA**. **a)** Fluorescence enhancement (upper panel) and fold increase over the mean value of initial readout at t = 0 (lower panel) of CDA or esterase-processed CDA (CDA, 10 µM; esterase, 10 µg/mL; 8 hour incubation) with *E. coli/*TEM-1 (10^6 c.f.u./mL) and H_2O_2 (1 mM). A.U. indicates arbitrary units. **b)** HPLC traces of CDA (10 µM) incubated with esterase (10 µg/mL). **c)** Mass spectra of peak β and γ in negative electrospray ionization modes.

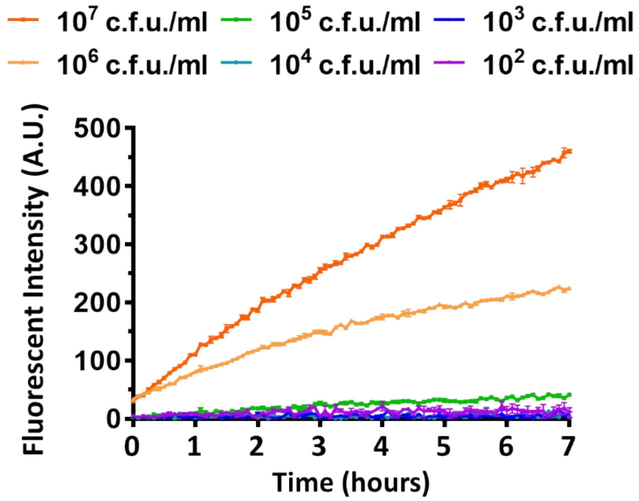

Figure S21. Fluorescence enhancement of CDA at different concentrations of *E. coli* expressing KPC-3 carbapenemase in PBS at room temperature; CDA, 10 μM, and H_2O_2, 1 mM. The signal of CDA in H_2O_2 was subtracted and the absolute values were plotted. Data were collected on a Tecan Safire microplate reader with excitation at 570 nm and emission at 600 nm. A.U. indicates arbitrary [illegible]

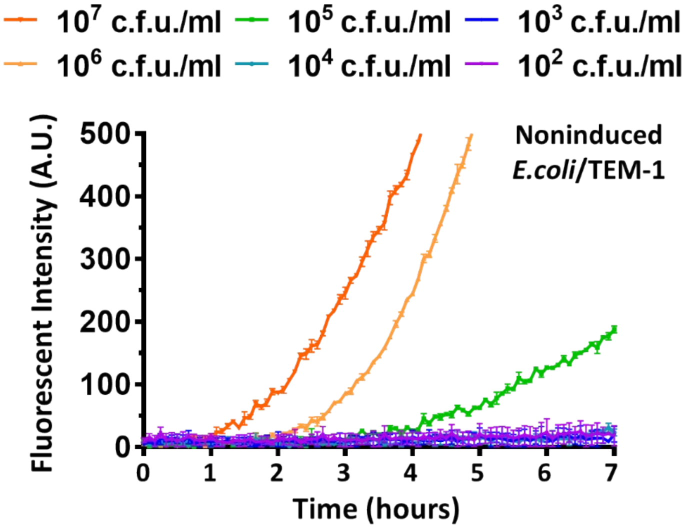

Figure S22. Fluorescence enhancement of CDA at concentrations of noninduced *E. coli*/TEM-1 in PBS at room temperature; CDA, 10 μM and H_2O_2, 1 mM. The signal of CDA in H_2O_2 was subtracted and the absolute values were plotted. Data were collected on a Tecan Safire microplate reader with excitation at 570 nm and emission at 600 nm. A.U. indicates arbitrary units.

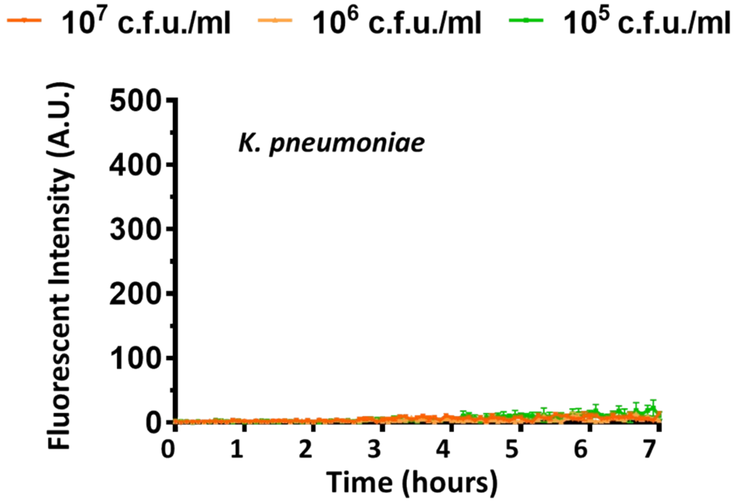

Figure S23. Fluorescence enhancement of CDA (10 μM) at different concentrations of imipenem sensitive *K. pneumoniae* in PBS at room temperature (H_2O_2, 1 mM). The signal of CDA in H_2O_2 was subtracted and the absolute values were plotted. Data were collected on a Tecan Safire microplate reader with excitation at 570 nm and emission at 600 nm. A.U. indicates arbitrary units.

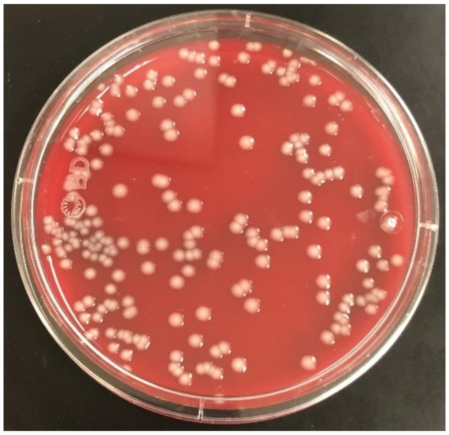

Figure S24. A representative blood agar plate with 220 colonies from 20 μL of 10^4 c.f.u./mL *K. pneumoniae* suspension determined by measuring its absorbance at OD_{600}.

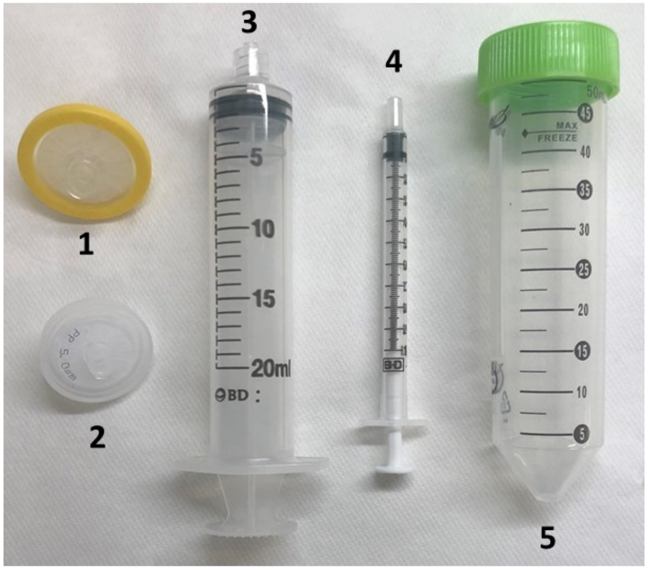

Figure S25. The kit used for urinary bacterial detection. The kit contains 1) a 0.22-µm polypropylene filter, 2) a 5-µm polypropylene filter, 3) a 20-mL sterile syringe, 4) a 1-mL sterile syringe, and 5) a 50-mL sterile conical tube.

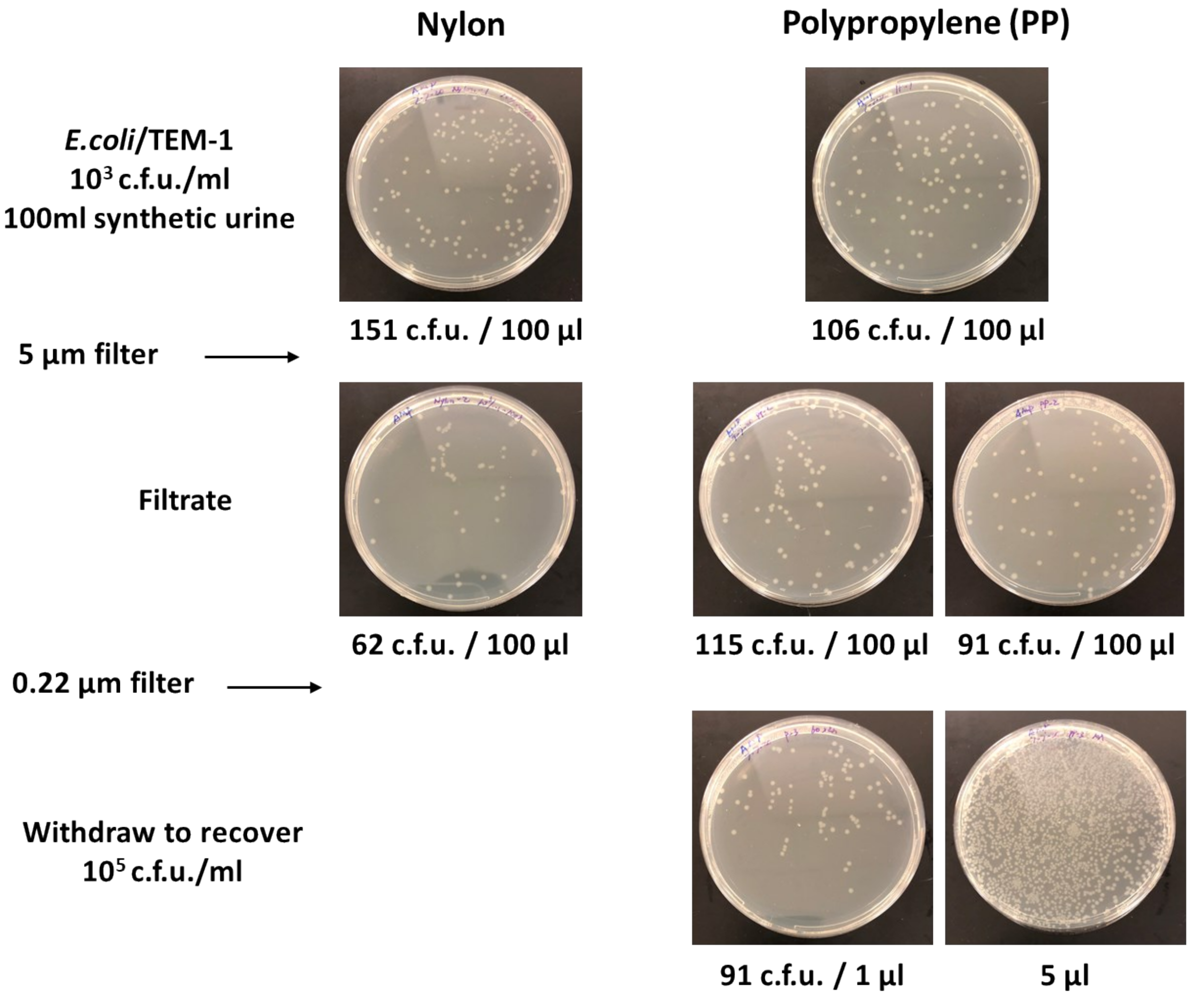

Figure S26. Comparison of nylon and polypropylene filters in concentrating bacteria from urine samples. Designated volume from 100 mL synthetic urine spiked with *E. coli*/TEM-1 Bla at 10^3 c.f.u./mL (top), filtrate after 5-µm filters (middle), and recovered bacteria in PBS (bottom) were spread on agar-based LB medium to monitor the efficiency of filtration and concentration.

References

1. Z. Han, X. Liang, X. Ren, L. Shang and Z. Yin, *Chemistry, an Asian journal*, 2016, **11**, 818-822.
2. H. A. Albrecht, G. Beskid, K. K. Chan, J. G. Christenson, R. Cleeland, K. H. Deitcher, N. H. Georgopapadakou, D. D. Keith, D. L. Pruess, J. Sepinwall and et al., *Journal of medicinal chemistry*, 1990, **33**, 77-86.
3. H. Shi, Y. Cheng, K. H. Lee, R. F. Luo, N. Banaei and J. Rao, *Angew. Chem. Int. Ed.*, 2014, **53**, 8113-8116.
4. J. Xie, X. Huang, M. S. Park, H. M. Pham and W. K. Chan, *Biochemical pharmacology*, 2014, **88**, 253-265.
5. T. Dai, J. Xie, Q. Zhu, M. Kamariza, K. Jiang, C. R. Bertozzi and J. Rao, *J. Am. Chem. Soc.*, 2020, **142**, 15259-15264.

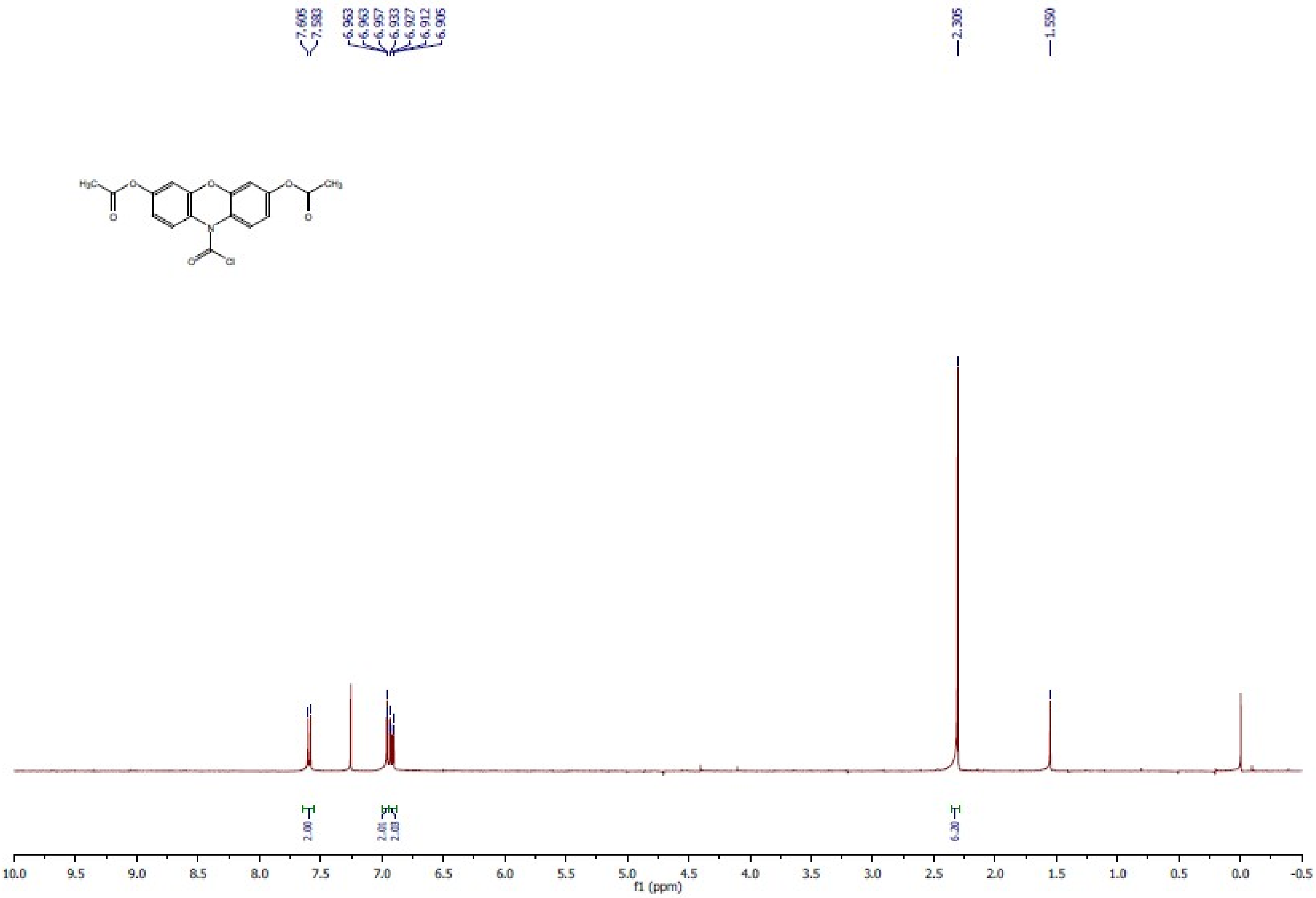

7.605
7.583
6.963
6.963
6.957
6.933
6.927
6.912
6.905
2.305
1.550
H3C
O
O
N
O
Cl
CH3
2.00
2.01
2.03
6.20
10.0 9.5 9.0 8.5 8.0 7.5 7.0 6.5 6.0 5.5 5.0 4.5 4.0 3.5 3.0 2.5 2.0 1.5 1.0 0.5 0.0 -0.5
f1 (ppm)

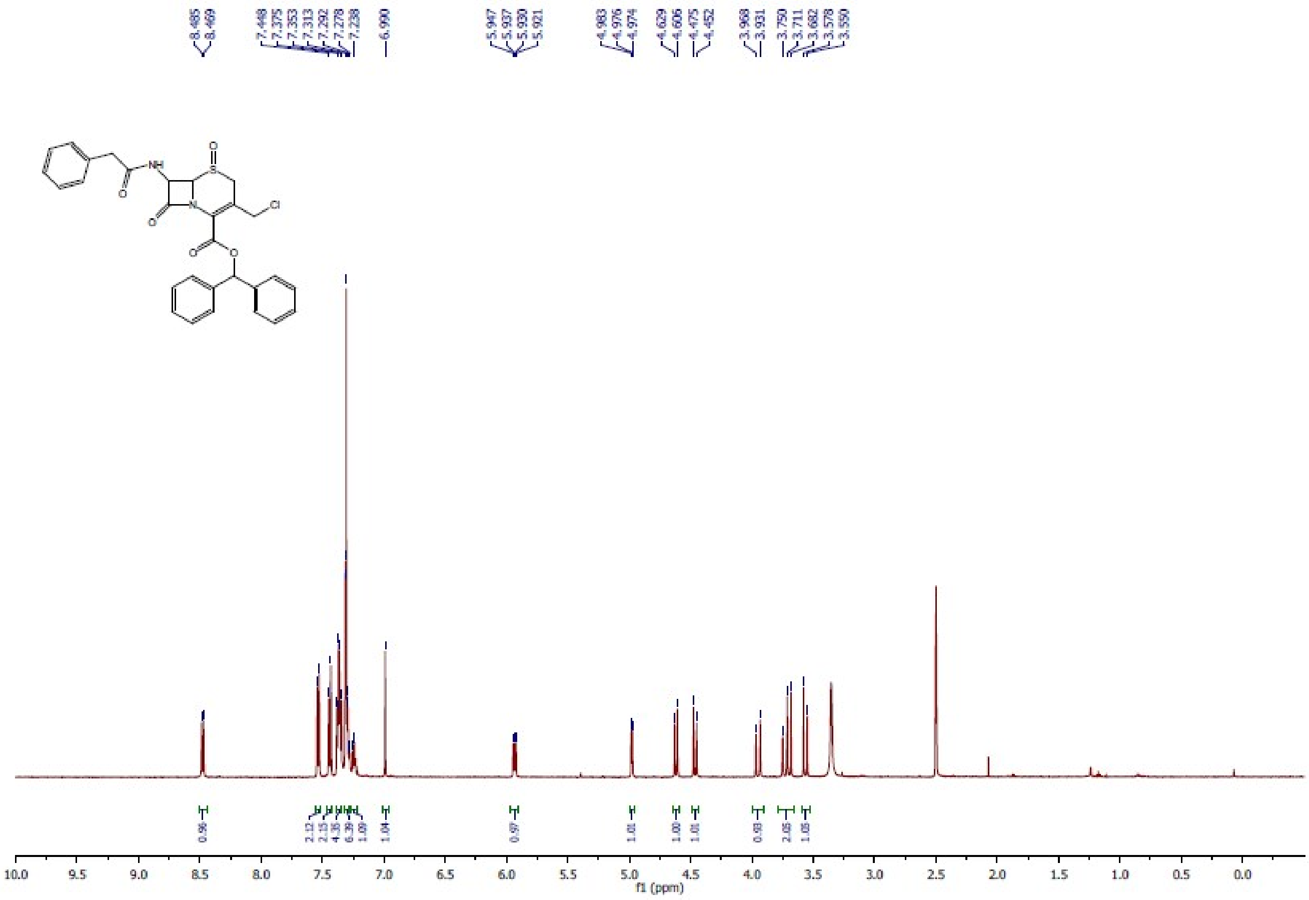

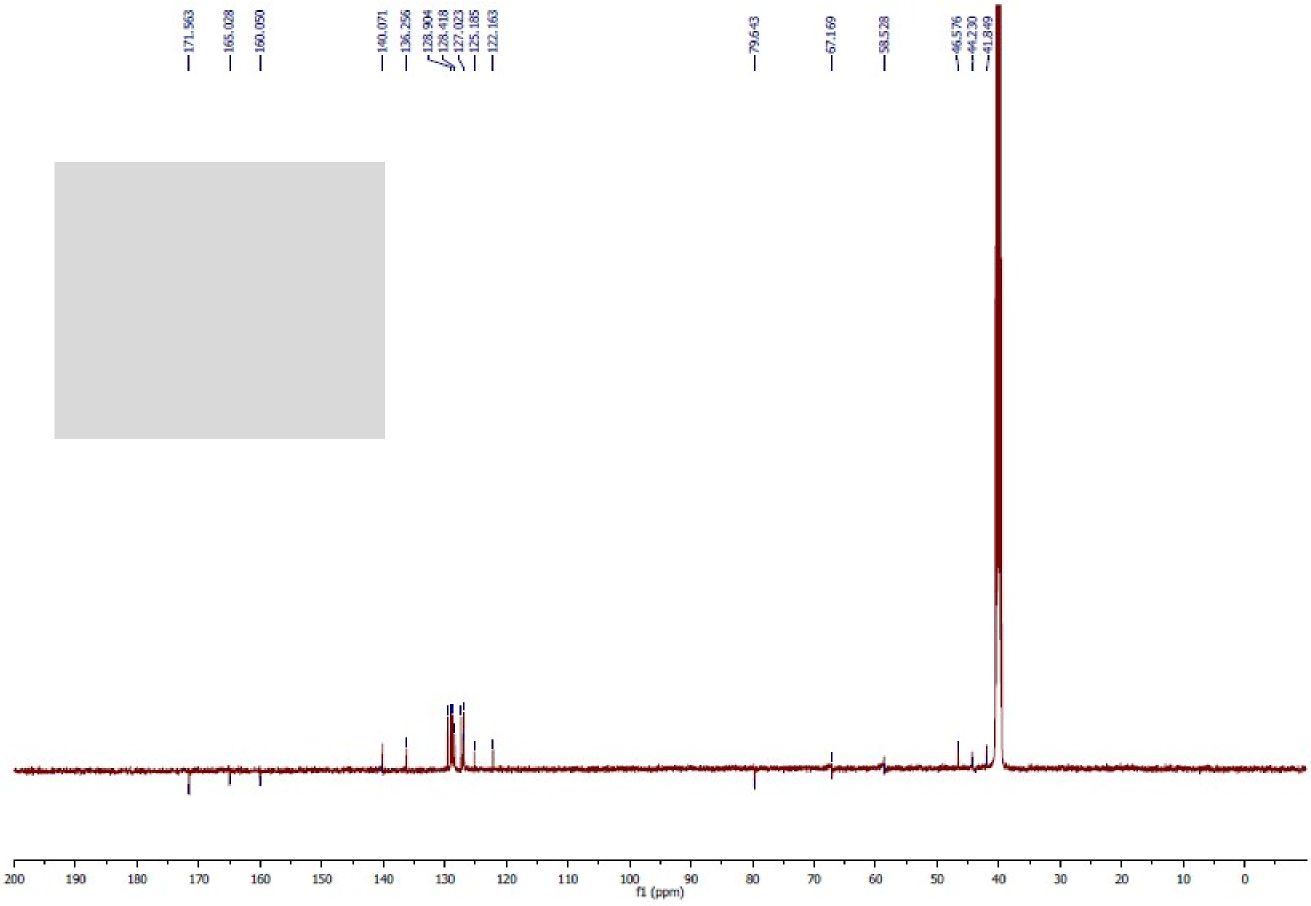
171.563
165.028
160.050
140.071
136.256
128.904
128.418
127.023
125.185
122.163
79.643
67.169
58.528
46.576
44.230
41.849
200 190 180 170 160 150 140 130 120 110 100 90 80 70 60 50 40 30 20 10 0
f1 (ppm)

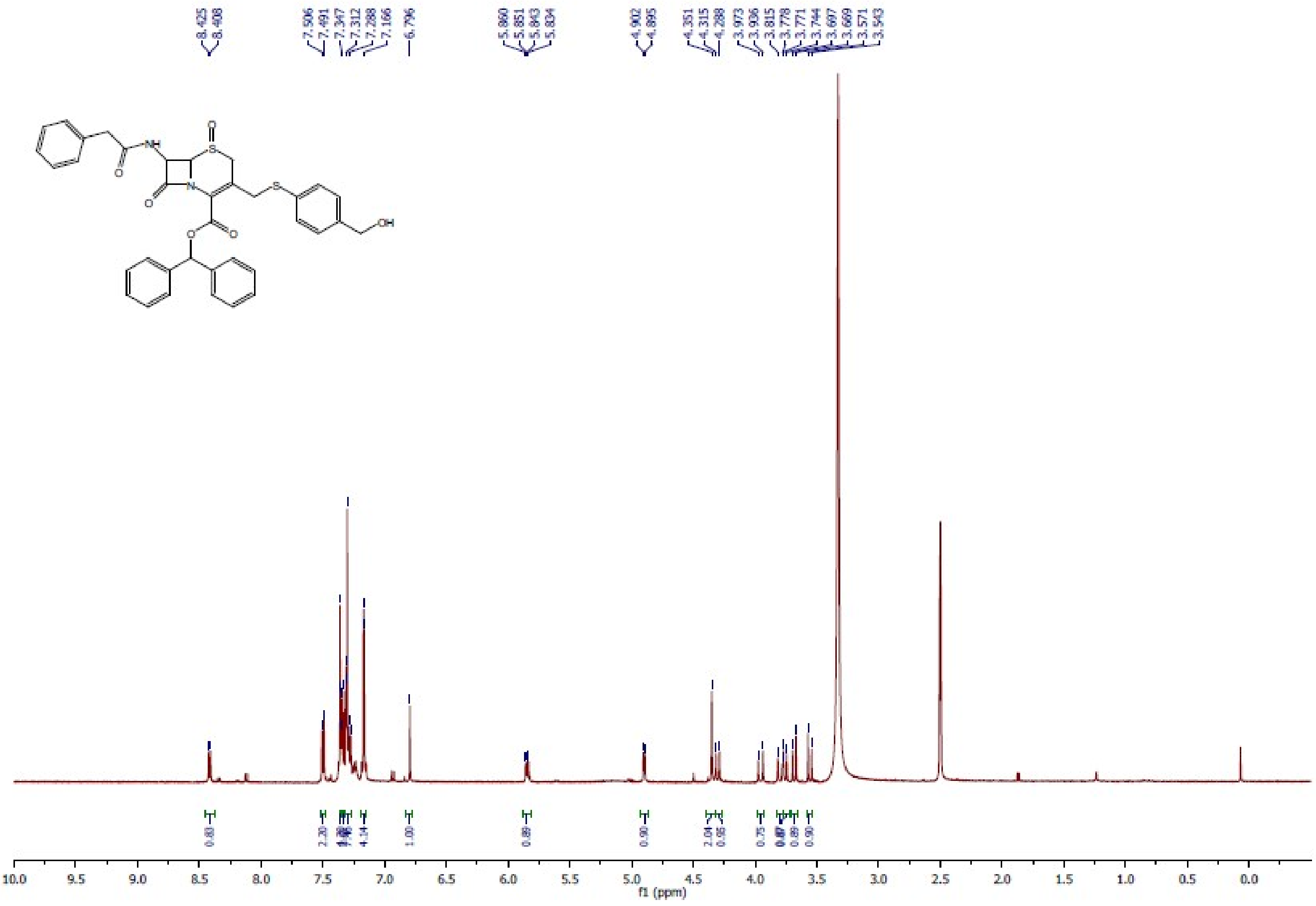
8.425
8.408
7.506
7.491
7.347
7.312
7.288
7.166
6.796
5.860
5.851
5.843
5.834
4.902
4.895
4.351
4.315
4.288
3.973
3.936
3.815
3.778
3.771
3.744
3.697
3.669
3.571
3.543
f1 (ppm)

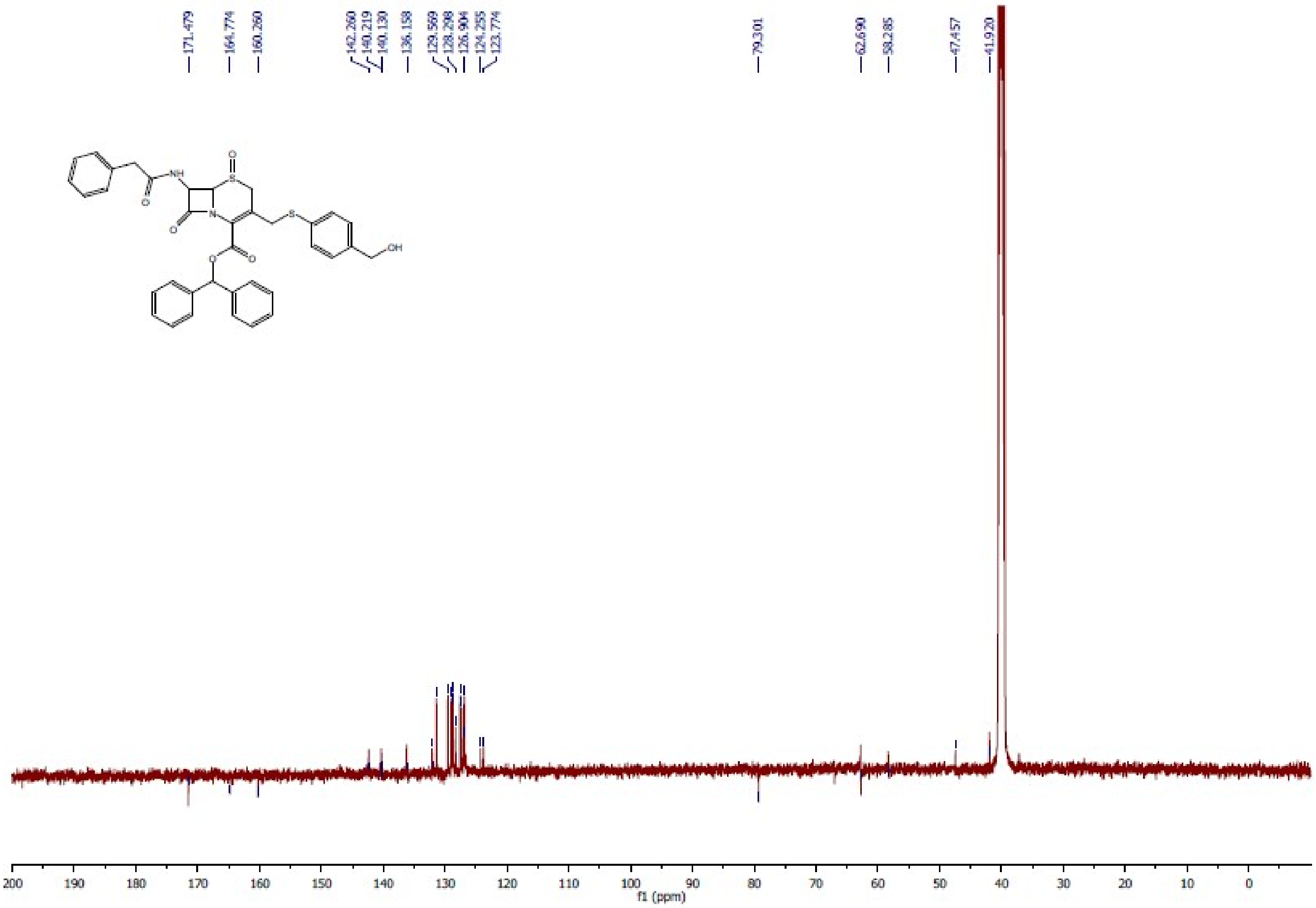
171.479
164.774
160.260
142.260
140.219
140.130
136.158
129.569
128.298
126.904
124.255
123.774
79.301
62.690
58.285
47.457
41.920
NH
S
O
N
OH
f1 (ppm)
200
190
180
170
160
150
140
130
120
110
100
90
80
70
60
50
40
30
20
10
0

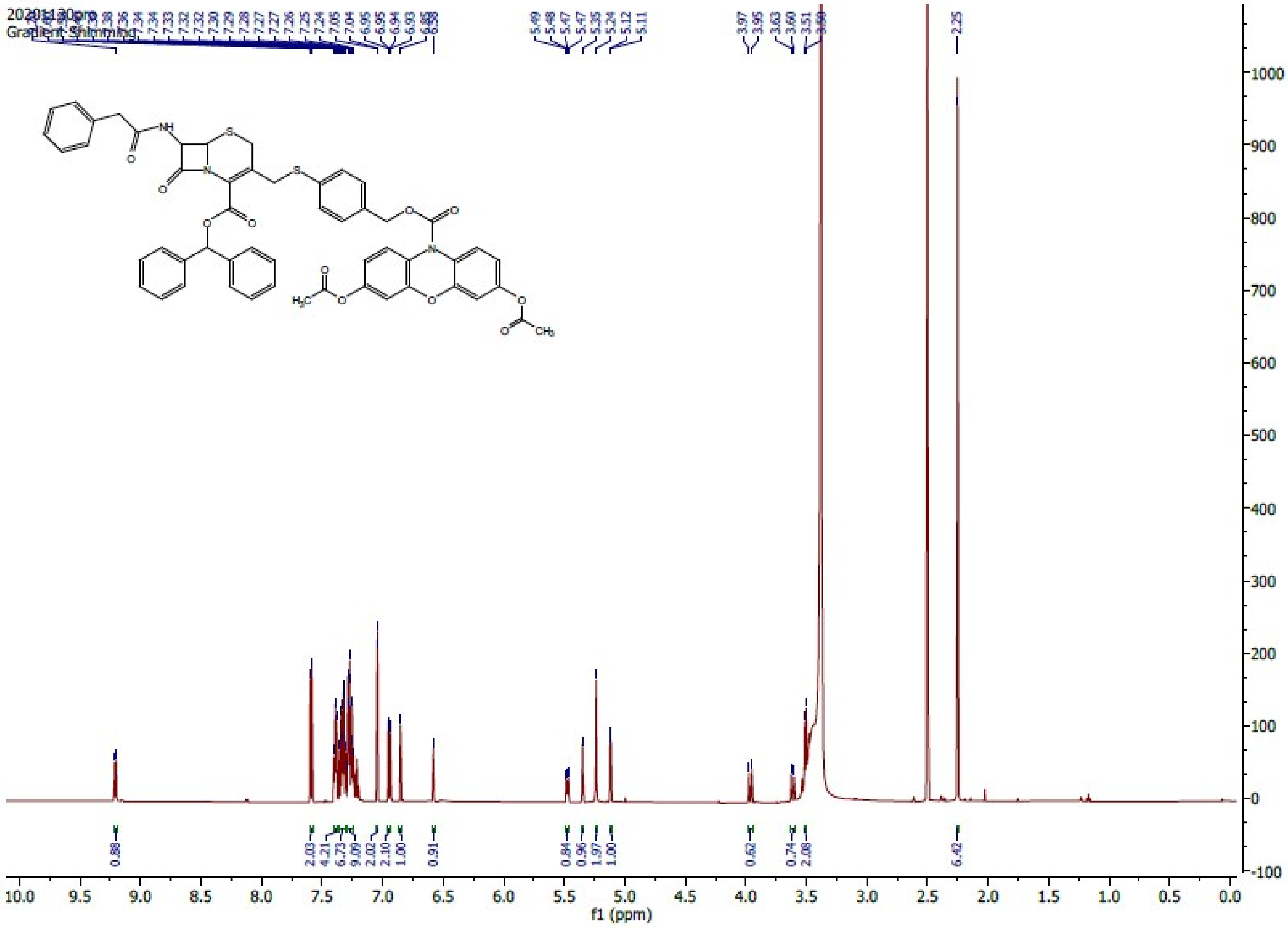

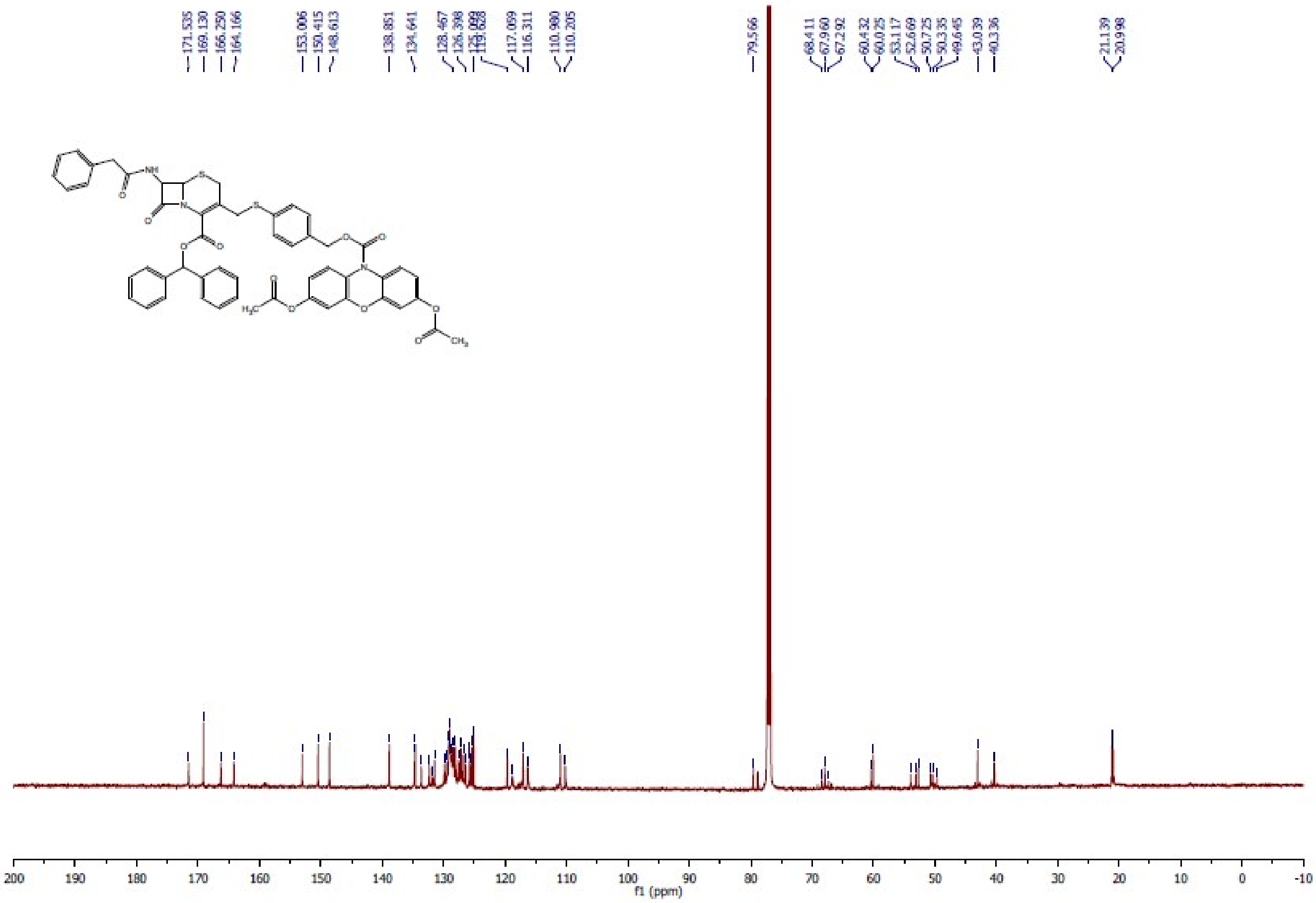
171.535
169.130
166.250
164.166
153.006
150.415
148.613
138.851
134.641
128.467
126.398
117.099
116.311
110.980
110.205
79.566
68.411
67.960
67.292
60.432
60.025
53.117
52.669
50.725
50.335
49.645
43.039
40.336
21.139
20.998
200
190
180
170
160
150
140
130
120
110
100
90
80
70
60
50
40
30
20
10
0
-10
f1 (ppm)

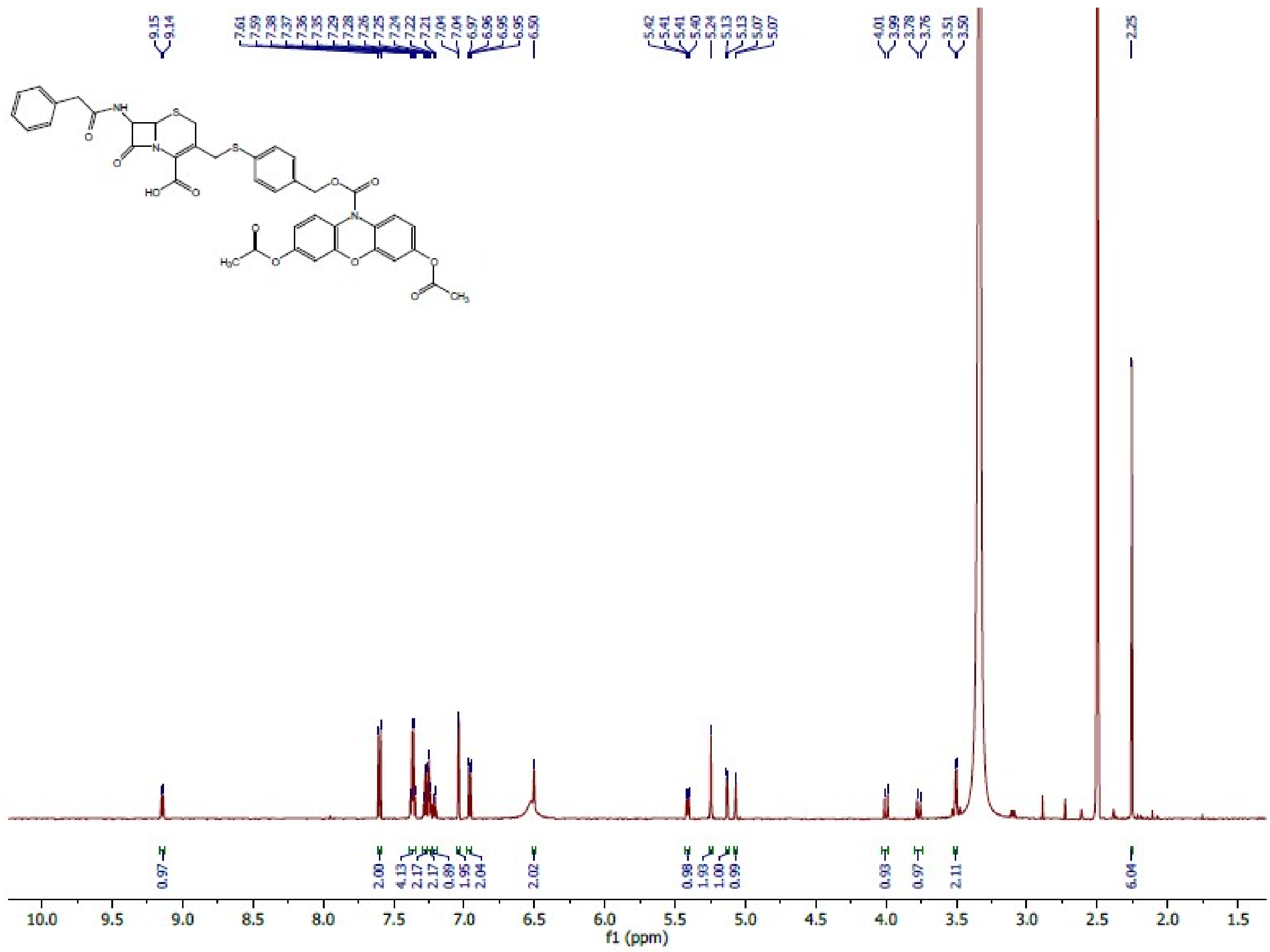

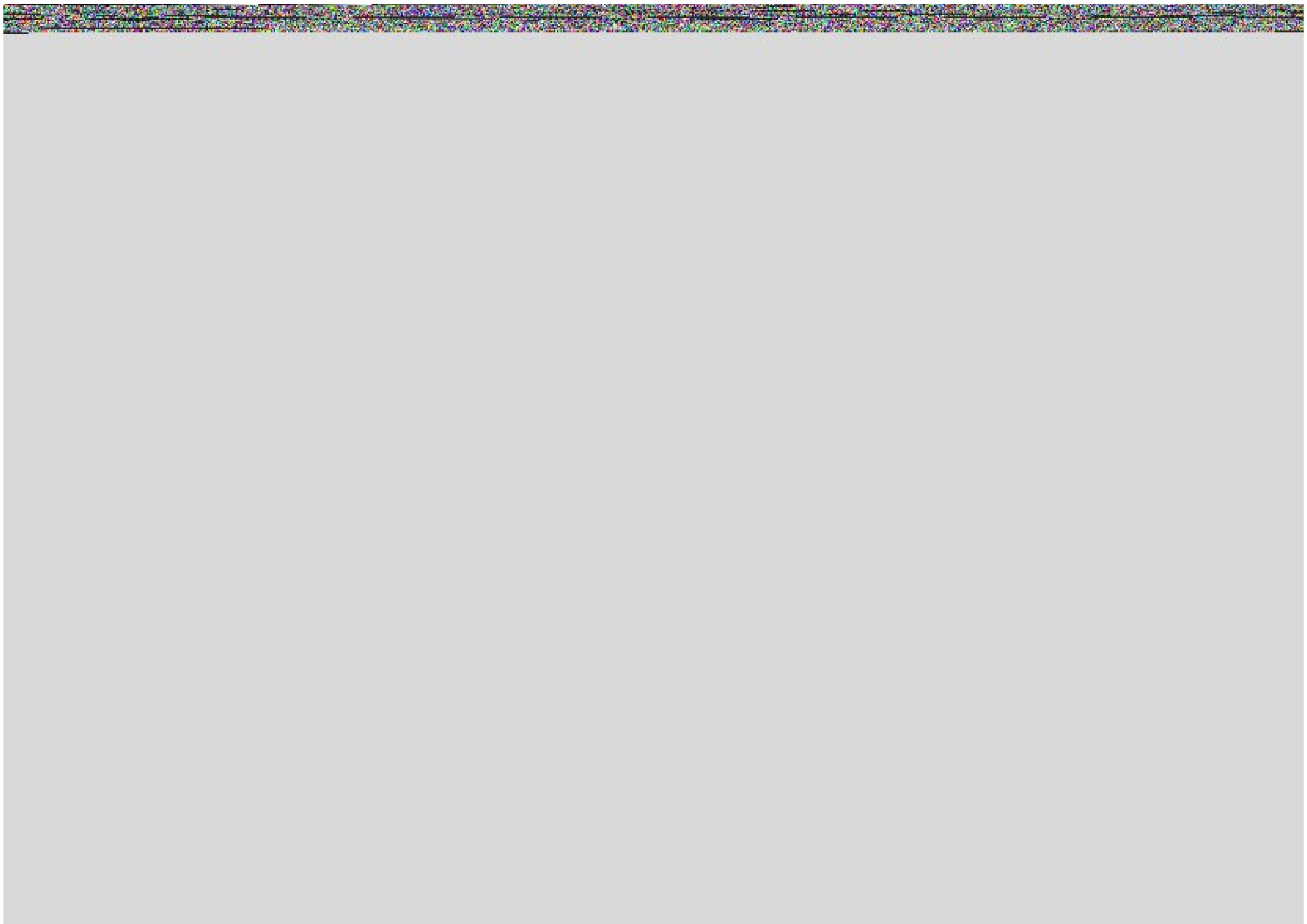

Supplement: SC-012-D1SC01471D-s001 [file SC-012-D1SC01471D-s001.pdf]
